# The FAP × CD3 bispecific antibody OMTX305 induces T cell–mediated antitumor effects in patient-derived ex vivo models of solid tumors

**DOI:** 10.1126/sciadv.aee2124

**Published:** 2026-09-11

**Authors:** Julia Thiel, Jan A. Schlegel, Chunguang Liang, Thomas E. Mürdter, Adrian Kneer, Sascha Dreher, Annelie Schäfer, Oliver Seifert, Lennart Kühl, Gerhard Preissler, Laureano Simon, Myriam Fabre, Isabel Egaña, Katrin S. Kurz, German Ott, Roland E. Kontermann, Walter E. Aulitzky, Matthias Schwab, Monilola A. Olayioye, Meng Dong

**Affiliations:** ^1^ Dr. Margarete Fischer-Bosch Institute of Clinical Pharmacology and University of Tübingen, 70376 Stuttgart, Germany.; ^2^ Institute of Immunology, Jena University Hospital, Friedrich-Schiller-University, 07743 Jena, Germany.; ^3^ Comprehensive Cancer Center Central Germany (CCCCG), Jena University Hospital, 07743 Jena, Germany.; ^4^ Department of Bioinformatics, Biocenter Am Hubland, University of Würzburg, 97074 Würzburg, Germany.; ^5^ Cluster of Excellence iFIT (EXC 2180) “Image-guided and Functionally Instructed Tumor Therapies”, University of Tübingen, Tübingen, Germany.; ^6^ Department of Gynecology and Obstetrics, Robert Bosch Hospital, 70376 Stuttgart, Germany.; ^7^ Department of Thoracic Surgery, Robert Bosch Hospital,70376 Stuttgart, Germany.; ^8^ Institute of Cell Biology and Immunology, University of Stuttgart, 70569 Stuttgart, Germany.; ^9^ Stuttgart Research Center Systems Biology (SRCSB), University of Stuttgart, 70569 Stuttgart, Germany.; ^10^ Oncomatryx Biopharma S.A., Derio 48160, Spain.; ^11^ Department of Clinical Pathology, Robert Bosch Hospital, 70376 Stuttgart, Germany.; ^12^ Department of Oncology, Robert Bosch Hospital, 70376 Stuttgart, Germany.; ^13^ German Cancer Consortium (DKTK), Partner Site Tübingen, German Cancer Research Center (DKFZ), Heidelberg, Germany.; ^14^ National Center for Tumor Diseases (NCT), NCT South-West, a partnership between DKFZ, University Hospital Tübingen, University of Tübingen, Tübingen, Bosch Health Campus, Stuttgart, Robert Bosch Hospital, Stuttgart, University Hospital Ulm, and Ulm University, Ulm, Germany.; ^15^ Departments of Clinical Pharmacology, and of Biochemistry and Pharmacy, University Tübingen, Tübingen, Germany.

## Abstract

Within solid tumors, stromal barriers and immune exclusion limit the success of immunotherapies. Thus, novel therapeutic approaches are required to remodel the tumor-supportive microenvironment and enhance therapeutic responses. We developed a bispecific, trivalent Fab-eIg T cell engager (OMTX305) targeting fibroblast activation protein (FAP), a marker of tumor-promoting cancer-associated fibroblasts. The translational potential of OMTX305 was investigated in 2D and 3D patient-derived preclinical models, with a focus on precision-cut tumor slices (PCTSs) of lung and ovarian cancer cocultured with autologous PBMCs. By combining spatial biology with cytokine and transcriptomic analysis, we show that OMTX305 treatment eliminated FAP-expressing fibroblasts, induced interferon responses and extracellular matrix remodeling, ultimately triggering bystander killing of adjacent tumor cells. Distinct immune phenotypes and PD-L1 expression patterns appeared to be associated with differential treatment responses, suggesting their potential as biomarkers for patient stratification. Our findings highlight the clinical potential of targeting stromal elements as a cancer therapy and establish PCTS as a powerful preclinical platform for personalized immunotherapy assessment.

## INTRODUCTION

Cancer-associated fibroblasts (CAFs) are key components of the tumor microenvironment (TME) of solid tumors that actively influence tumor growth, invasion, and therapy resistance. Far from being passive structural elements, CAFs secrete cytokines, growth factors, and extracellular matrix proteins that promote angiogenesis, modulate immune cell infiltration, and facilitate tumor cell survival. Through reciprocal signaling with malignant and immune cells, they help establish an immunosuppressive and protumorigenic niche. The heterogeneity and plasticity of CAF populations further underscore their multifaceted roles in cancer progression. Consequently, targeting CAFs and their interactions within the TME has become an emerging therapeutic strategy aimed at disrupting the supportive stromal network that sustains tumor development (*1*).

Elevated fibroblast activation protein (FAP) expression has been observed in 90% of carcinomas and is associated with a poor prognosis in different malignancies, including high-grade serous ovarian cancer (HGSOC) and non–small cell lung cancer (NSCLC) (*2*–*4*). Because the cell-surface serine protease is predominantly expressed by CAFs (*4*, *5*), targeting FAP therapeutically is a promising approach to remodel the tumor stroma and re-establish tumor immunity.

Bispecific molecules, particularly T cell engagers (TCEs), are a promising class of immunotherapies, with more than 100 TCEs investigated in clinical studies (*6*). TCEs are designed to bind both CD3 on T cells and a target-specific antigen on cancer cells or the TME, thereby redirecting T cells to a malignant target (*6*). Only upon simultaneous binding of the two antigens, an immunological synapse is formed, leading to T cell activation and target cell lysis through the release of cytolytic granules and pro-inflammatory cytokines (*7*–*9*). While TCEs are highly successful in hematologic malignancies, their clinical success in solid tumors remains limited (*10*). Among the primary barriers is the immunosuppressive TME of solid tumors, restricting T cell infiltration and cytotoxic function (*10*, *11*). Here, we developed a novel bispecific, trivalent antigen-binding fragment engineered immunoglobulin (Fab-eIg) TCE targeting FAP and CD3 (OMTX305), aiming to modulate the TME and restore T cell functionality. We systematically evaluated this strategy, starting from two-dimensional (2D) cultured matched patient-derived CAF and normal fibroblasts (NFs), progressing to more complex 3D CAF spheroids and heterotypic CAF-tumor cell spheroids, and ultimately focusing on precision-cut tumor slices (PCTSs) derived from patients with ovarian and lung cancer as advanced model systems. Our results demonstrate that OMTX305 treatment can activate T cells to induce target cell death in FAP-positive stromal regions and bystander killing of adjacent tumor cells. Notably, the results of PCTS suggest to further study immune phenotypes in HGSOC and immune checkpoint profiles in NSCLC as predictors of response to OMTX305 therapy and patient stratification. Together, our findings underscore the therapeutic potential of stromal-targeting strategies in cancer. This study further highlights PCTS as a translational ex vivo platform to profile cell type–specific treatment responses within the native TME, offering a promising avenue toward personalized immunotherapy.

## RESULTS

### OMTX305 induces FAP-dependent cytotoxicity in patient-derived CAFs via T cell engagement

**Fig. 1.**
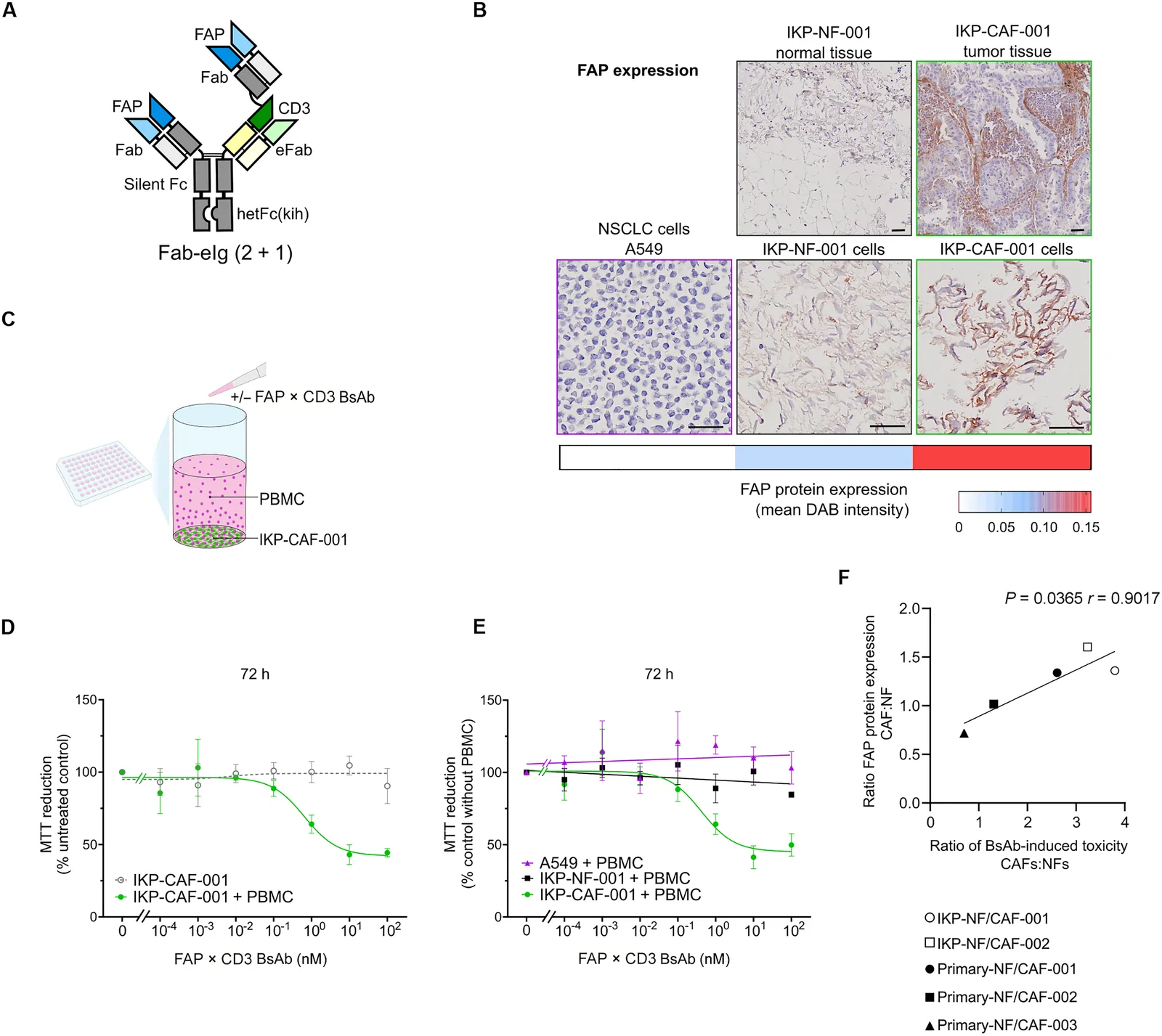
FAP × CD3 BsAb (OMTX305) reduces the viability of FAP-expressing fibroblasts cocultured with PBMCs. (**A**) Composition of the 2 + 1 Fab-eIg TCE. (**B** to **F**) Immortalized CAF/NF (IKP-CAF/NF-001) were derived from NSCLC tumor tissue and adjacent normal lung tissue. (B) FAP protein expression in IKP-CAF/NF-001 cells or original tissue and in A549 NSCLC tumor cells, shown by and quantified from IHC staining. (means ± SD of three areas with >1000 cells per area). Scale bars, 50 μm. (C) Cocultures with allogenic PBMCs were OMTX305-treated. (D) MTT assay showing relative viability of OMTX305-treated IKP-CAF-001 with or without PBMC coculture (*n* = 3, means ± SD). (E) MTT assay showing relative viability of A549, IKP-CAF-001 or IKP-NF-001 cocultured with PBMCs and OMTX305 treated (*n* = 3, means ± SD). (F) Correlation of relative OMTX305-induced toxicity to FAP expression in CAF/NF pairs.

We first generated immunoglobulin G–like bispecific FAP × CD3 targeting TCEs by using the previously established eIg platform technology (*12*), characterized either by a 1 + 1 (eIg) or 2 + 1 (Fab-eIg) stoichiometry (Fig. 1A and fig. S1A). Purified molecules showed the expected heavy and light chain bands in SDS–polyacrylamide gel electrophoresis analysis and a single peak in analytical size exclusion chromatography (fig. S1, B and C). Notably, the presence of two FAP-binding sites in the 2 + 1 Fab-eIg translated into a strongly increased binding to FAP-expressing HT1080-FAP cells compared to that of the 1 + 1 eIg [EC_50_ (median effective concentration) values of 0.03 nM for the Fab-eIg nad 14 nM for the eIg] (fig. S1D). No binding was seen with FAP-negative HT1080 cells, confirming specificity (fig. S1E). Both molecules were capable of binding to CD3^+^ Jurkat cells, although here the 2 + 1 Fab-eIg showed a ∼26-fold weaker binding compared to the 1 + 1 eIg (EC_50_ values of 46 nM for the Fab-eIg and 1.3 nM for the eIg) (fig. S1H). Nevertheless, compared to the eIg, the Fab-eIg mediated a 20-fold increased killing of the HT1080-FAP target cells in the presence of peripheral blood mononuclear cells (PBMCs) from healthy donors (EC_50_ values of 0.15 nM for the Fab-eIg and 3.1 nM for the eIg in fig. S1F). No cytotoxicity was observed with HT1080 wild-type (WT) cells, demonstrating the selective killing of cells with ectopic FAP expression (fig. S1G). Fab-eIg further more potently stimulated CD4^+^ and CD8^+^ T cell proliferation and activation of T cells, as measured by interferon-γ (IFN-γ) release (fig. S1, I to K). In conclusion, the trivalent 2 + 1 Fab-eIg molecule (designated OMTX305) showed superior T cell engaging activity and was thus used for further studies. To exclude OMTX305 binding to tissues expressing baseline levels of FAP, tissue cross-reactivity studies were conducted with the same anti-FAP scFv sequence. No specific staining was observed in human nontarget tissues (fig. S1, L to N, and table S3), suggesting a lack of detectable OMTX305 binding to healthy human tissues.

Next, we evaluated the efficacy of OMTX305 in primary fibroblasts endogenously expressing different levels of FAP, as these cells represent the intended target of the TCE. Therefore, CAFs and NFs were isolated from the tumor and adjacent normal tissue of a patient with NSCLC, generating a pair of immortalized cell lines, termed IKP-CAF-001 and IKP-NF-001, as described previously (*13*). Immunohistochemistry (IHC) analysis of the original patient tissue confirmed FAP expression in the tumor stroma, while adjacent normal tissue lacked detectable FAP expression (Fig. 1B). Consistently, IKP-CAF-001 expressed higher FAP protein and mRNA levels than IKP-NF-001, whereas the NSCLC tumor cell line A549 showed no detectable FAP mRNA or protein expression (Fig. 1B and fig. S2D).

To test the cells sensitivity to OMTX305, we set up a coculture of CAFs with allogeneic PBMCs from healthy donors (Fig. 1C). The coculture induced a baseline alloreactive cytotoxicity (fig. S2, A to C). Similarly to HT1080-FAP cells (fig. S1D), the exposure to OMTX305 decreased the viability of FAP^+^ IKP-CAF-001 dose dependently, but only in the presence of PBMCs (Fig. 1D). In contrast, the viability of FAP^−^ A549 and IKP-NF-001 was unaffected by OMTX305 treatment (Fig. 1E), confirming target-specific cytotoxicity, most likely mediated by T cell engagement.

We then analyzed one additional immortalized patient-derived CAF/NF pair (fig. S2, D to G) and three primary CAF/NF pairs (fig. S2, H to P). A variable FAP expression was observed in these isolated CAF/NF pairs, as well as their original tissues (figs. S2 and S3). In NF, FAP levels were up-regulated by the ex vivo culture, in line with the culture-induced phenotypic drift of fibroblasts reported previously (*14*, *15*). The TCE-mediated cytotoxicity closely correlated to the relative difference in FAP protein expression within each CAF/NF pair (Fig. 1F). In summary, we found that OMTX305 treatment reduces fibroblast viability of CAFs in a FAP- and immune cell–dependent manner.

### FAP-expressing CAF spheroids are infiltrated and killed by T cells in response to OMTX305

**Fig. 2.**
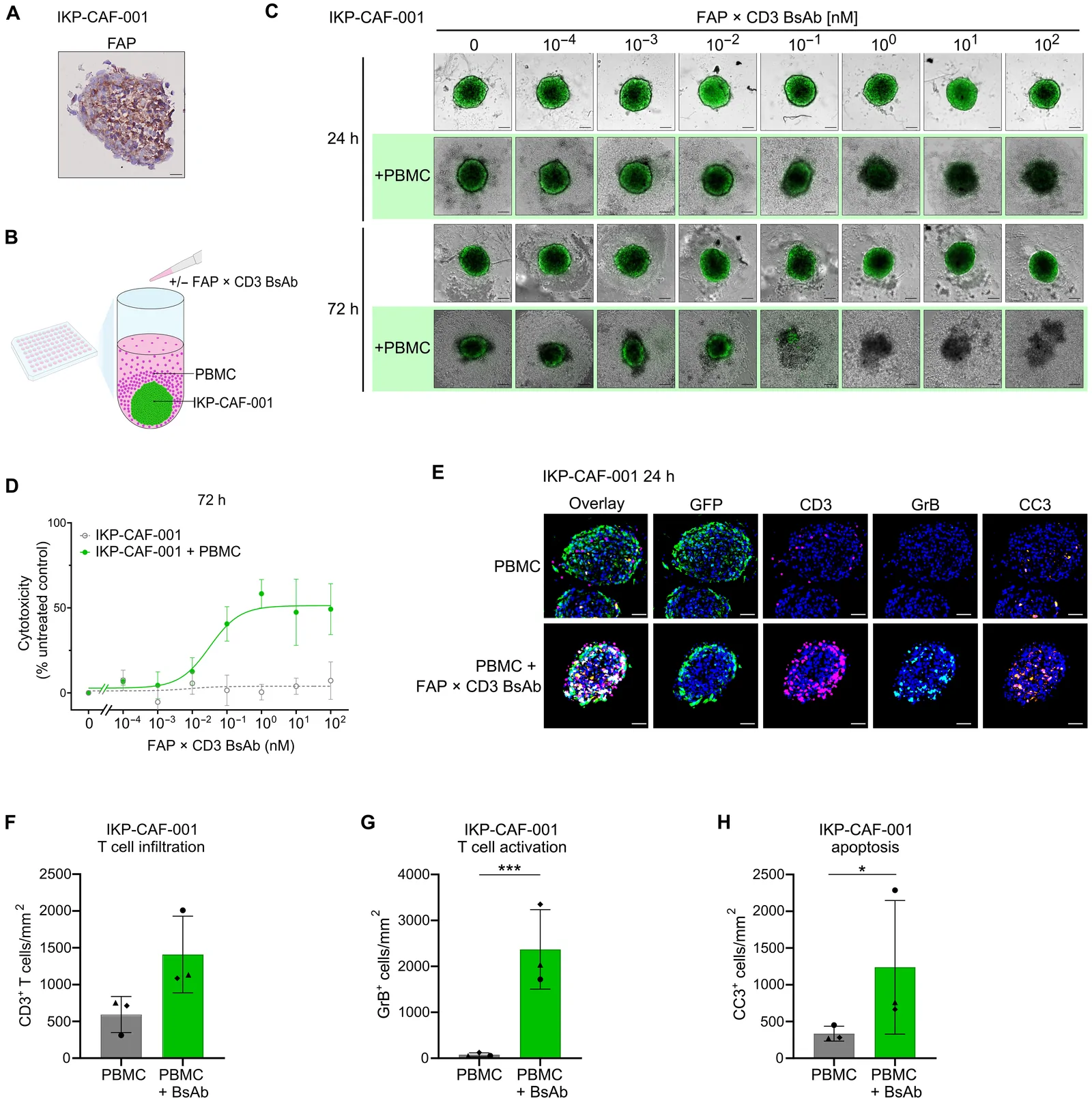
FAP × CD3 BsAb (OMTX305) recruits T cells to induce apoptosis in FAP-expressing CAF spheroids. (**A**) FAP expression of IKP-CAF-001 spheroids shown by IHC staining. Scale bar, 50 μm. (**B**) Experimental setup of OMTX305 treatment of GFP^+^ IKP-CAF-001 spheroids cocultured with allogeneic PBMCs. (**C** to **H**) GFP-expressing IKP-CAF-001 spheroids were cocultured with PBMCs and OMTX305-treated for up to 72 hours. (C) Overlay of fluorescence and brightfield images of IKP-CAF-001 spheroids treated with increasing BsAb concentrations. Scale bars, 200 μm. (D) BsAb-induced cytotoxicity in IKP-CAF-001 spheroids with or without PBMC coculture, determined by lactate dehydrogenase quantification in the supernatant (*n* = 3, means ± SD). (E) T cell infiltration, activation, and apoptosis induction in IKP-CAF-001 spheroids cocultured with PBMCs and treated with 1 nM OMTX305 for 24 hours detected by mIF staining of nuclei (DAPI, blue), CAFs (GFP, green), T cells (CD3, pink), T cell activation (GrB, cyan), and apoptosis (CC3, orange). Scale bars, 50 μm. (F to H) T cell infiltration and biomarker expression in IKP-CAF-001 spheroids quantified from mIF stainings showing the (F) T cell density (CD3), (G) T cell activation (GrB), and (H) apoptosis induction (CC3) (*n* = 3, mean of 10 spheroids per experiment ± SD) (two-tailed ratio paired *t* test; *P ≤ 0.05 and ***P ≤ 0.001).

We next investigated whether OMTX305 treatment kills CAFs and promotes T cell infiltration into 3D cell structures. We therefore generated spheroids of green fluorescent protein (GFP)–labeled IKP-CAF-001, which maintained their FAP protein expression in 3D culture (Fig. 2A). Spheroids were cocultured with allogeneic PBMCs from healthy donors and treated with OMTX305 (Fig. 2B). In the presence of PBMCs, OMTX305 treatment induced a dose-dependent cytotoxicity, characterized by the destruction of the overall structure, loss of GFP^+^ cells, and increased LDH release (Fig. 2, C and D). To assess T cell infiltration and activation in IKP-CAF-001 spheroids, we performed mIF stainings (Fig. 2E). After 24 hours of coculture, CD3^+^ T cells penetrated into CAF spheroids with significantly enhanced infiltration following OMTX305 treatment (T cell density: 593 cells/mm^2^ before treatment and 1409 cells/mm^2^ after treatment) (Fig. 2F). On the basis of the expression of granzyme B (GrB), infiltrating T cells were significantly activated by the treatment (Fig. 2G) and induced apoptosis in IKP-CAF-001 cells, as revealed by cleaved caspase-3 (CC3) staining (Fig. 2H). To confirm the FAP dependency of these effects, we repeated the spheroid assays with the NSCLC tumor cell line H1437, which lacks FAP expression (fig. S4A). Here, OMTX305 treatment induced neither T cell infiltration, activation, nor cytotoxicity (fig. S4, B to G). In summary, we demonstrated that OMTX305 promotes T cell infiltration, activation, and target cell apoptosis in 3D FAP-expressing cell structures.

### In heterotypic spheroids, OMTX305 induces cell death in CAFs and tumor cells

**Fig. 3.**
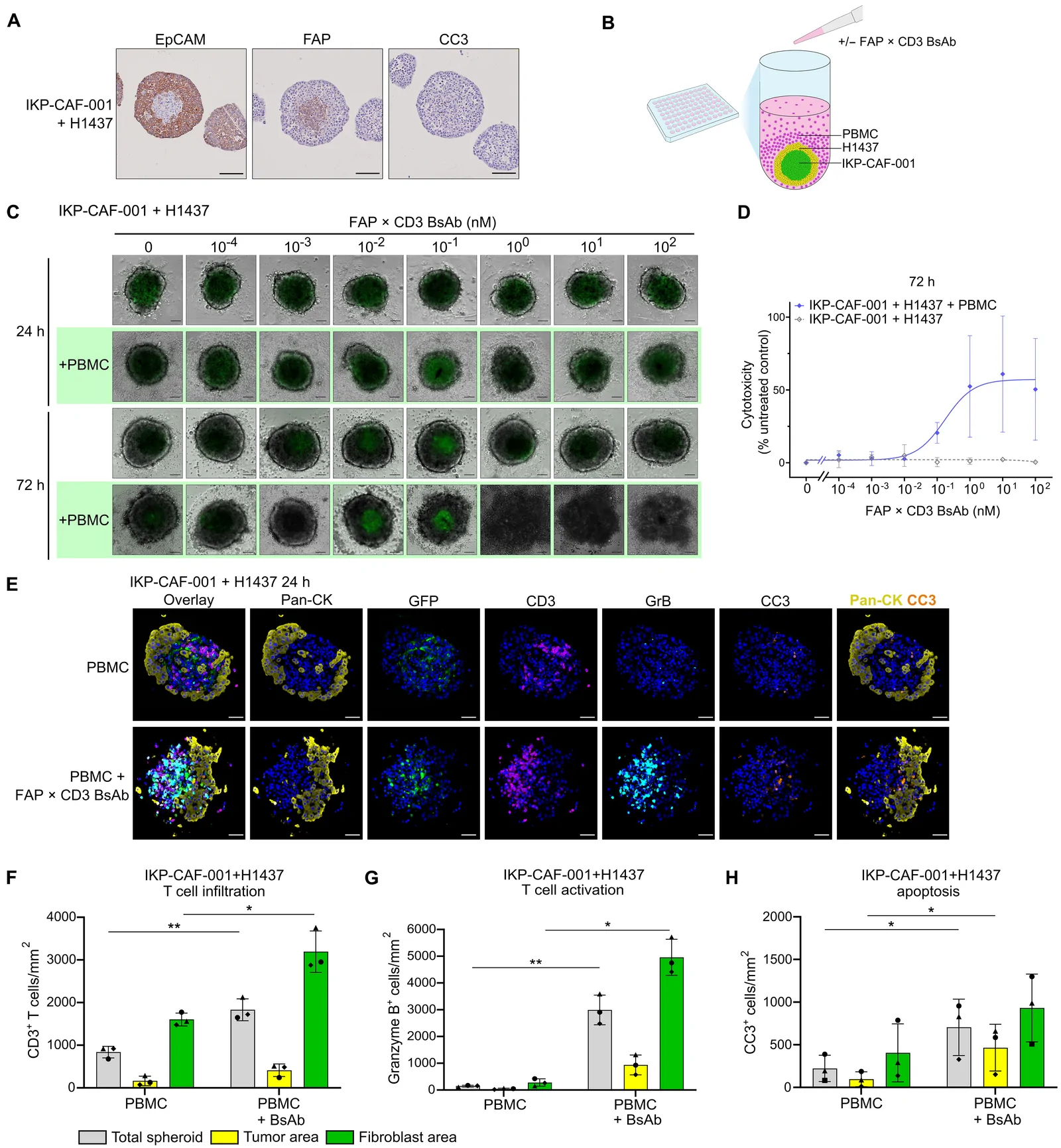
CAFs and tumor cells are both affected by FAP × CD3 BsAb (OMTX305) treatment in heterotypic spheroids. (**A**) IHC staining of heterotypic spheroids composed of H1437 cells (EpCAM^+^) in the border and IKP-CAF-001 (FAP^+^) in the spheroid core. Viability shown by CC3-staining. Scale bars, 100 μm. (**B**) Experimental setup of OMTX305 treatment of heterotypic spheroids cocultured with allogeneic PBMCs. (**C**) Overlay of fluorescence and bright-field images of heterotypic spheroid and PBMC cocultures treated with increasing OMTX305 concentrations. Scale bars, 200 μm. (**D**) OMTX305-induced cytotoxicity in heterotypic spheroids with or without PBMC coculture. Cytotoxicity determined by lactate dehydrogenase quantification in the supernatant (*n* = 3, means ± SD). (**E**) T cell infiltration, activation, and apoptosis induction in heterotypic spheroids cocultured with PBMCs and treated with 1 nM FAP × CD3 BsAb detected by mIF staining of nuclei (DAPI, blue), tumor cells (pan-CK, yellow), fibroblasts (GFP, green), T cells (CD3, pink), T cell activation (GrB, cyan), and apoptosis (CC3, orange). Scale bars, 50 μm. (**F** to **H**) Quantification of mIF stainings showing (F) T cell density (CD3), (G) T cell activation (GrB), and (H) apoptosis induction (CC3) in heterotypic spheroids (*n* = 3, means of 10 spheroids per experiment ± SD) (two-tailed ratio paired t test; *P ≤ 0.05 and **P ≤ 0.01).

To mimic the in vivo tumor complexity more closely, we generated heterotypic spheroids comprising FAP^+^ fibroblasts and FAP^−^ tumor cells, allowing us to track the response of both cell types to OMTX305 treatment. Heterotypic spheroids were established by co-seeding GFP-expressing IKP-CAF-001 and H1437 NSCLC tumor cells in a 3:1 ratio. In these spheroids, the core consisted of FAP^+^ fibroblasts (IKP-CAF-001), which were surrounded by EpCAM^+^ H1437 tumor cells. Under baseline conditions, heterotypic spheroids were viable and showed no signs of apoptosis, indicated by the absence of CC3 (Fig. 3A). Upon coculture with PBMCs and OMTX305 treatment (Fig. 3B), spheroids showed a time- and dose-dependent structural disintegration, loss of the GFP signal (Fig. 3C), and increased cytotoxicity (Fig. 3D). To assess the spatial dynamics of T cell infiltration, activation, and apoptosis, we performed mIF stainings (Fig. 3E). The tumor area was defined by a pan-cytokeratin (pan-CK) staining of H1437 tumor cells, while GFP expression marked the fibroblast compartment. Independent of treatment, allogeneic T cells infiltrated from the surrounding PBMC coculture into the fibroblast core (Fig. 3, E and F). In the absence of OMTX305, infiltrated T cells were not activated (Fig. 3, E and G). OMTX305 treatment significantly enhanced the T cell accumulation and activation, with increased CD3 density and GrB expression in the fibroblast core, alongside a nonsignificant trend toward a higher apoptosis (CC3) induction (Fig. 3, F to H). Tumor cell apoptosis was increased significantly following treatment. This indicates that targeting FAP^+^ fibroblasts with OMTX305 not only enhances immune infiltration and cytotoxicity within the fibroblast compartment but also promotes apoptosis in neighboring tumor cells, suggesting a bystander antitumor effect.

### T cells are activated by OMTX305 in PCTS

**Fig. 4.**
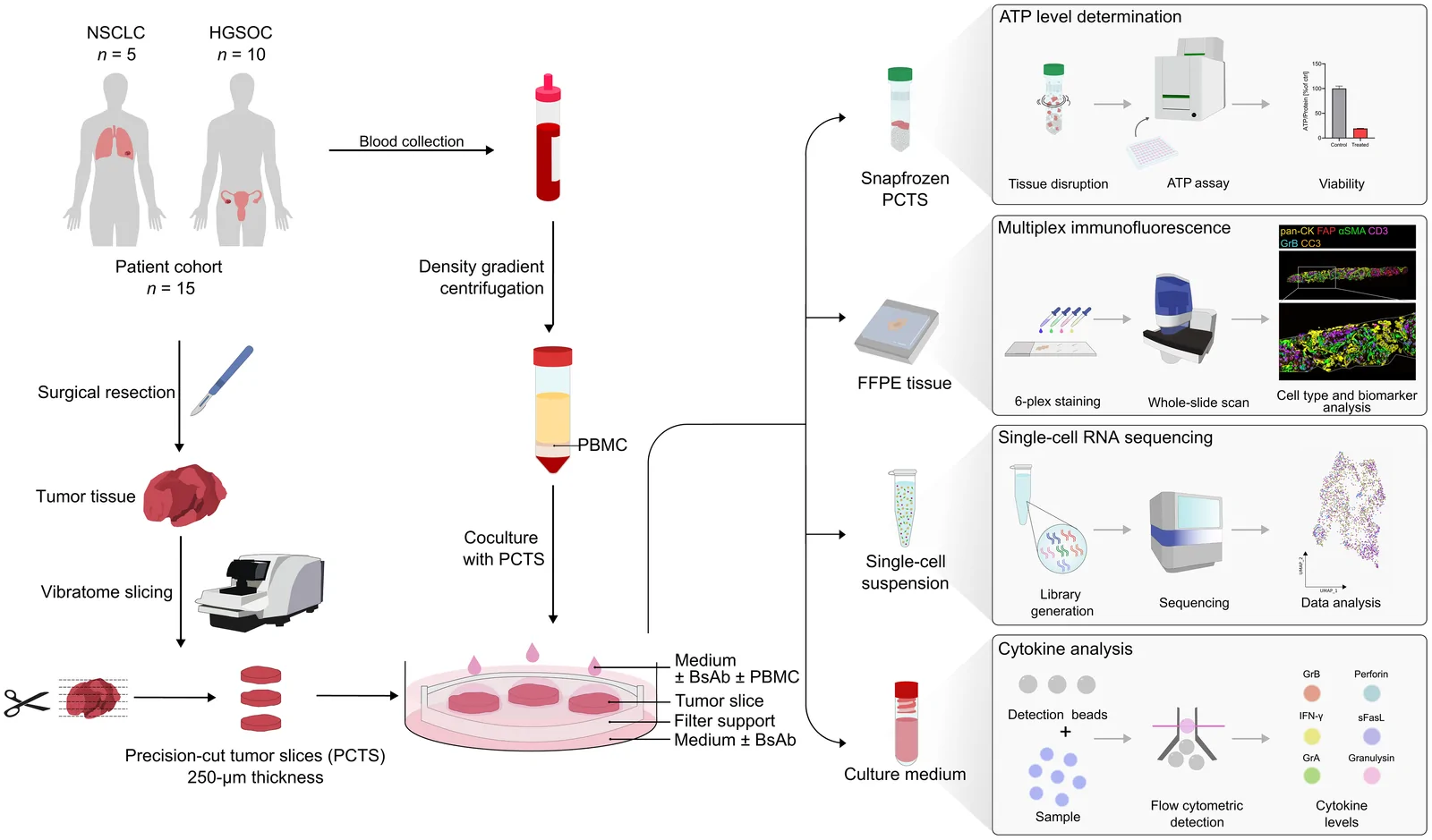
Workflow of individual drug response assessment in PCTS. Human NSCLC or HGSOC tissue was sliced into 250-μm thick PCTS using a vibratome on the day of surgical resection. Autologous PBMCs were isolated from patient blood. PCTS were OMTX305-treated on a filter support with or without PBMC coculture for up to 3 days. Harvested PCTSs were disrupted for ATP-based viability determination, FFPE-embedded for mIF stainings, or processed for single-cell RNA-seq. Cytokines were determined from the supernatant using a flow cytometry–based assay.

We next evaluated the therapeutic potential of OMTX305 in PCTS, which more closely recapitulate the structural and cellular complexity of in vivo tumors. OMTX305 treatment responses were investigated in PCTS derived from 15 primary human tumors, comprising 5 NSCLC and 10 HGSOC samples (Fig. 4). To this end, PCTS were treated with OMTX305 with or without PBMC coculture for up to 3 days. Treatment responses were assessed by adenosine 5′-triphosphate (ATP)–based viability assays, cytokine profiling in culture supernatants, and mIF stainings, which allow the tracking of treatment responses within different cell types (fig. S5, A and B). Responses of CAF subsets were further monitored by single-cell RNA sequencing (single-cell RNA-seq). The ability of cocultured autologous T cells to infiltrate into PCTS was verified by 3D imaging using confocal microscopy (fig. S5C). In the absence of OMTX305, coculture with autologous PBMCs did not induce T cell activation or cytotoxicity in PCTS (fig. S5, D to G).

**Fig. 5.**
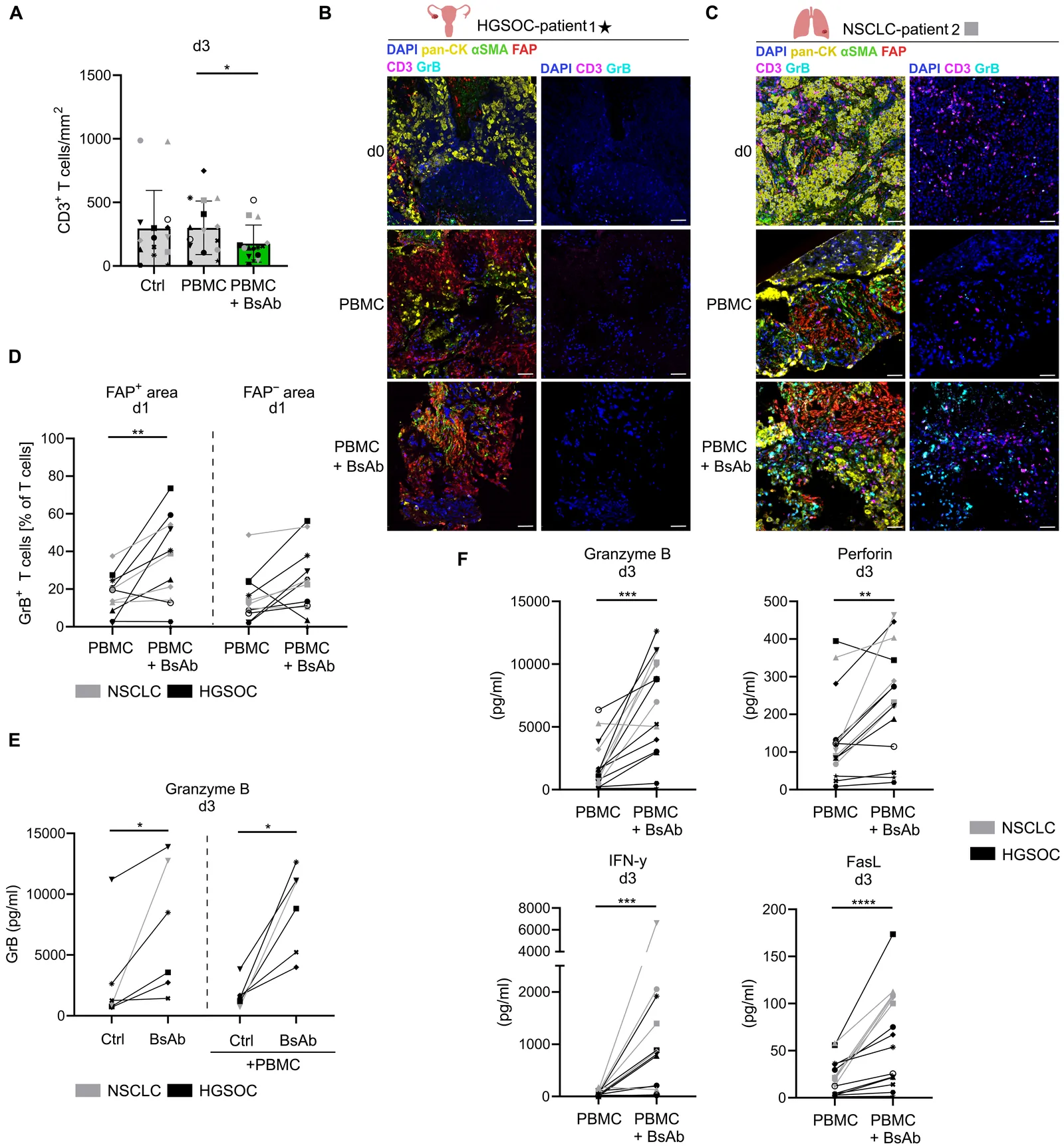
FAP × CD3 BsAb (OMTX305) activates tissue-resident T cells in PCTS. (**A** to **F**) PCTS (total *n* = 15) of NSCLC (*n* = 5) or HGSOC (*n* = 10) tumors were OMTX305-treated for up to 3 days with or without PBMC coculture. Different patients are indicated by different symbols and colors. (A) T cell density in untreated (ctrl) PCTS or PCTS cocultured with PBMCs with or without BsAb (Wilcoxon signed-rank test; *P ≤ 0.05). (B) Absence of T cells and Granzyme B (GrB) in HGSOC-patient 1 (indicated by a black star) detected by mIF staining of T cells (CD3, pink), GrB (cyan), tumor cells (pan-CK, yellow), fibroblasts (αSMA, green; FAP, red), and nuclei (DAPI, blue). Scale bar, 50 μm. Minor presence of GrB in this patient can also be seen in (F). (C) T cell activation in NSCLC-patient 2 shown by mIF staining as explained in (B). Scale bars, 50 μm. NSCLC-patient 2 is indicated as a grey square. (D) T cell activation in FAP^+^ or FAP^−^ areas quantified from mIF stainings for CD3 (T cells), GrB, and FAP (Wilcoxon signed-rank test; **P ≤ 0.01). (E) GrB levels in the PCTS culture supernatant with or without PBMC coculture (*n* = 6) (Wilcoxon signed-rank test; *P ≤ 0.05). (F) GrB, Perforin, IFN-γ, or FasL levels in the supernatant (Wilcoxon signed-rank test; **P ≤ 0.01, ***P ≤ 0.001, and ****P ≤ 0.0001).

To first determine whether OMTX305 treatment promotes infiltration of T cells from cocultured autologous PBMCs into tumor slices, we quantified CD3^+^ T cell densities from mIF stainings. While CD3^+^ T cell density was not affected by PBMC coculture (Ctrl: 294 T cells/mm^2^, PBMC: 300 T cells/mm^2^), T cell density was significantly reduced to 175 cells/mm^2^ after 3 days of OMTX305 treatment (Fig. 5A). In one case (HGSOC-patient 1) neither the baseline tumor tissue (d0), nor the untreated control PCTS showed T cell infiltration, indicating that treatment with OMTX305 did not induce detectable T cell recruitment in this patient (Fig. 5B). However, in T cell–infiltrated cases, mIF stainings revealed a significant increase in GrB^+^ T cells within the FAP^+^ stromal area following 1 day of treatment (Fig. 5, C and D). T cell activation was further demonstrated by cytokine profiling of the PCTS culture supernatant after 3 days of treatment, showing a significant, treatment-induced increase in GrB and Perforin production (Fig. 5, E and F). An OMTX305-dependent increase in cytolytic cytokines was also observed in the absence of cocultured PBMCs, indicating a localized activation of preexisting tissue-resident T cells (Fig. 5E and fig. S6, A and B). While no significant difference in T cell activation was observed between OMTX305 treatment of PCTS based on the presence of cocultured PBMCs, cytotoxic activity, reflected by GrB levels in the supernatant, was elevated in four of the six patient PCTSs when cocultured with PBMCs (Fig. 5E). Notably, OMTX305 treatment further led to a significant increase in other cytolytic proteins including Granzyme A and Granulysin, as well as the pro-inflammatory cytokine IFN-γ and the tumor necrosis factor family member Fas ligand (FasL) (Fig. 5F and fig. S6, C to F). In summary, we demonstrate that OMTX305 can activate tissue-resident T cells within patient-derived PCTS, resulting in the release of cytolytic and immunostimulatory mediators.

### Cell death is induced in FAP-expressing fibroblasts and adjacent tumor cells in PCTS

**Fig. 6.**
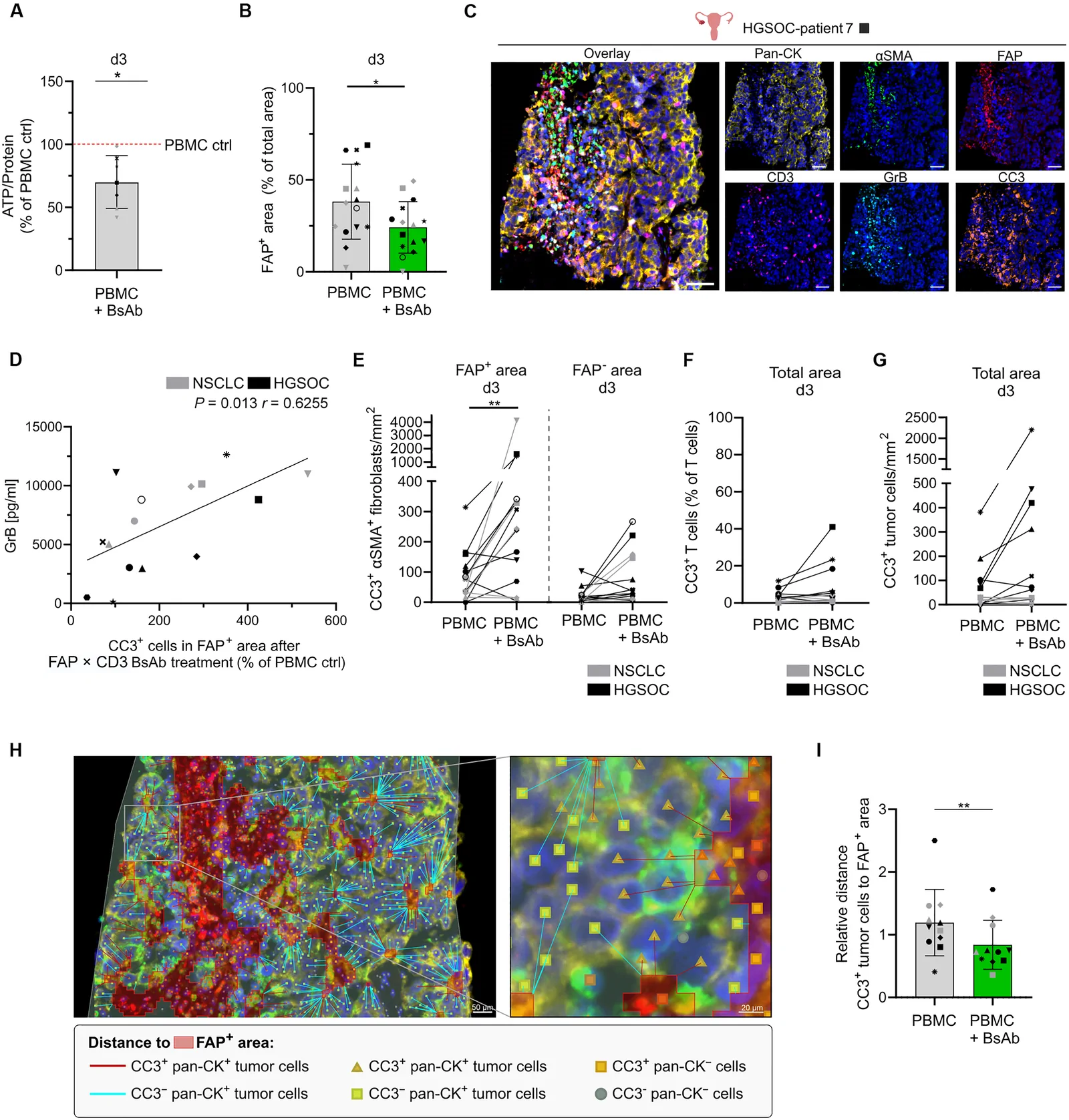
Cell death induction in fibroblasts and tumor cells in response to FAP × CD3 BsAb (OMTX305) correlates to the patient-specific T cell activation in PCTS. (**A** to **G**) PCTS (total *n* = 15) of NSCLC (*n* = 5) or HGSOC (*n* = 10) tumors were cocultured with autologous PBMCs and OMTX305-treated for up to 3 days. Different patients are indicated by different symbols and colors. (A) ATP levels reflecting PCTS viability (*n* = 7) (One sample Wilcoxon signed-rank test; *P ≤ 0.05). (B) Mean percentage of FAP^+^ PCTS area (Wilcoxon signed-rank test; *P ≤ 0.05). (C) Apoptosis induction in HGSOC-patient 7 detected by mIF staining of T cells (CD3, pink), Granzyme B (GrB, cyan), apoptosis (CC3, orange), tumor cells (pan-CK, yellow), fibroblasts (αSMA, green; FAP, red), and nuclei (DAPI, blue). Scale bars, 50 μm. HGSOC-patient 7 is indicated by a black square. (D) Pearson correlation between GrB cytokine levels in the supernatant and CC3^+^ cells in the FAP^+^ region. (E) CC3^+^ fibroblast density (CC3^+^ αSMA^+^) in FAP^+^ and FAP^−^ regions (Wilcoxon signed-rank test **P ≤ 0.01). (F) Percentage of apoptotic T cells (% CC3^+^ of CD3^+^) in the total PCTS area. (G) CC3^+^ tumor cell density (CC3^+^ pan-CK^+^) in the total PCTS area. (**H**) Schematic representation of distance analysis between CC3^+^ tumor cells (CC3^+^ pan-CK^+^) and the FAP^+^ region. Data were normalized to distances between CC3^−^ tumor cells (CC3^−^ pan-CK^+^) and the FAP^+^ region (*n* = 15). (**I**) Normalized distance of CC3^+^ tumor cells to FAP^+^ area (Wilcoxon signed-rank test; **P ≤ 0.01).

Given the significant activation of tissue-resident T cells observed in PCTS following OMTX305 treatment, we next assessed whether immune activation translated into target cell death. PCTS viability was assessed by an ATP-based assay and mIF analysis. OMTX305 treatment significantly reduced ATP levels to 69.6% compared to PBMC-only controls (Fig. 6A). Consistent with this, we observed a significant decrease in the proportion of FAP^+^ area from 38.2 to 24.2% following treatment (Fig. 6B). CC3^+^ staining revealed apoptosis induction in response to OMTX305 treatment for 3 days (Fig. 6C), which strongly correlated with T cell activation, as measured by GrB levels in the PCTS culture supernatants (Fig. 6D).

To address which cell types were affected by OMTX305-induced apoptosis, we analyzed colocalization of CC3 with lineage markers using mIF. A significant increase in apoptosis was detected in αSMA^+^ fibroblasts in the FAP^+^ area. In contrast, fewer αSMA^+^ fibroblasts were CC3^+^ in FAP^−^ areas (Fig. 6E), supporting the target dependency of the cytotoxic effect. In some patients, this was accompanied by an increase in CC3^+^ T cells (Fig. 6F). In addition, a subset of patient samples showed an elevated density of apoptotic pan-CK^+^ tumor cells following OMTX305 treatment (Fig. 6G).

The magnitude of OMTX305-dependent cell death induction in αSMA^+^ fibroblasts, CD3^+^ T cells, and pan-CK^+^ tumor cells was comparable between PCTS with or without PBMC coculture, suggesting that cytotoxicity was primarily mediated by tissue-resident T cells (fig. S7). Similar to the effects observed on T cell activation, coculture with PBMCs increased the density of CC3^+^ fibroblasts in two of the six patients (one NSCLC and one HGSOC), as well as the densitiy of CC3^+^ tumor cells in two patients with HGSOC (fig. S7, A and C).

To investigate whether tumor cell apoptosis was spatially linked to FAP^+^ regions, we performed a distance analysis of CC3^+^ versus CC3^−^ tumor cells relative to FAP^+^ areas. Tumor cells within the FAP^+^ area were assigned a distance of zero. The visualization of this analysis is shown in (Fig. 6H). We found that CC3^+^ tumor cells were located significantly closer to FAP^+^ areas than CC3^−^ tumor cells, suggesting a potential bystander killing effect (Fig. 6I). On average, apoptotic tumor cells were located 10 to 100 μm from the FAP^+^ area (table S4). In conclusion, these findings demonstrate that OMTX305 treatment induces apoptosis in FAP-expressing fibroblasts within patient-derived PCTS and, in a fraction of cases, elicits secondary tumor cell death in regions adjacent to FAP^+^ stroma.

The analysis of individual patient-derived PCTS revealed an interpatient variability of the response to OMTX305 treatment (fig. S8). In particular, one tumor lacking T cell infiltration (HGSOC-patient 1) showed no significant response of GrB or CC3 after exposure to OMTX305. In contrast, tumors with preexisting CD3^+^ T cell infiltration generally displayed T cell activation, shown by an increased GrB expression (e.g., HGSOC-patients 3 and 7). However, the extent of downstream cytotoxic effects varied. Similar heterogeneity was observed in NSCLC samples (fig. S8).

### Potential impact of immune phenotypes and immune exhaustion on OMTX305 response

**Fig. 7.**
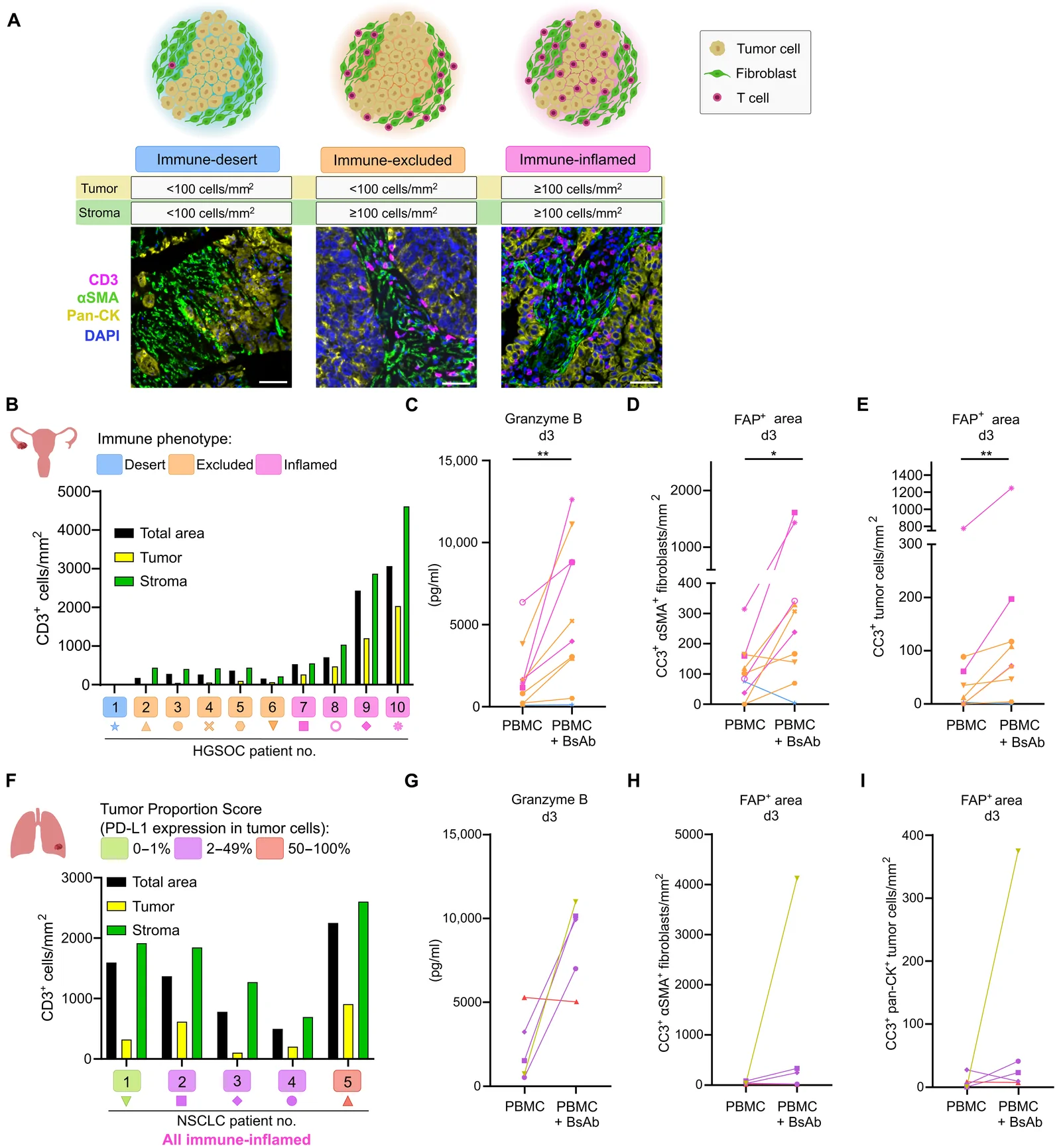
Immune phenotypes and PD-L1 expression patterns may predict FAP × CD3 BsAb (OMTX305) treatment responses in PCTS. (**A**) Tumors were grouped according to their immune phenotypes: immune-desert (minimal T cell presence), immune-excluded (T cells in stroma, but excluded from tumor), and immune-inflamed (T cells infiltrating both tumor and stroma). Scale bars, 50 μm. (**B**) Tumor immune phenotypes of HGSOC patient samples, classified according to (A). (**C** to **E**) HGSOC PCTS-PBMC cocultures (*n* = 10) were OMTX305-treated for 3 days. Experiments are colored by immune-phenotypes. (C) GrB levels in the PCTS supernatant (Wilcoxon signed-rank test; **P ≤ 0.01). (D) CC3^+^ fibroblast (CC3^+^ αSMA^+^) density in the FAP^+^ area (Wilcoxon signed-rank test *P ≤ 0.05). (E) CC3^+^ tumor cell (CC3^+^ pan-CK^+^) density in the FAP^+^ area (Wilcoxon signed-rank test **P ≤ 0.01). (**F**) Tumor immune phenotypes of NSCLC patient samples, classified according to (A). All samples were immune-inflamed and subclassified by the tumor cell PD-L1 expression. (**G** to **I**) NSCLC PCTS-PBMC cocultures (*n* = 5) were OMTX305-treated for 3 days. Experiments are colored by PD-L1 expression. (G) GrB levels in the PCTS supernatant. (H) CC3^+^ fibroblast (CC3^+^ αSMA^+^ cells) density in the FAP^+^ area. (I) CC3^+^ tumor cell (CC3^+^ pan-CK^+^ cells) density in the FAP^+^ area.

On the basis of our observations regarding the influence of T cell distribution on OMTX305 treatment efficacy, we hypothesized that, similarly to other immunotherapies (*16*, *17*), the baseline immune phenotype of a tumor may predict its OMTX305 therapy response. To investigate this, we stratified patients into three immune phenotypes based on the CD3^+^ T cell localization in the original (d0) tumor tissue: immune-desert tumors with minimal T cell presence, immune-excluded tumors with T cell infiltration restricted to the stroma, and immune-inflamed tumors with T cells infiltrating both the tumor and stroma (Fig. 7A). The cutoffs for each phenotype were set to 100 cells/mm^2^ based on our patient cohort (*18*, *19*).

Among the HGSOC cohort, all three immune phenotypes were represented: one immune-desert, five immune-excluded, and four immune-inflamed cases (Fig. 7B). The immune-desert patient (HGSOC-patient 1, blue) lacked T cells and consequently did not show GrB production or apoptosis induction in response to OMTX305 treatment (Fig. 7, C to E). In contrast, immune-inflamed tumors (HGSOC-patients 7 to 10, pink), mostly displayed among the highest levels of T cell activation and apoptosis induction. Immune-excluded tumors (HGSOC-patients 2 to 6) showed variable responses, with a spectrum of GrB release and apoptosis induction. Notably, immune-excluded and immune-inflamed HGSOC samples responded to OMTX305 treatment with cell death induction in both fibroblasts and tumor cells in the FAP^+^ region.

In the NSCLC cohort, although tumors expressed different ratios of T cell infiltration in the tumor and stroma area, all samples were classified as immune-inflamed and had an overall higher T cell density than HGSOC patients (Fig. 7F). In addition, treatment responses varied, suggesting that additional factors may influence sensitivity to OMTX305. In clinical practice, eligibility for immunotherapy in patients with NSCLC is guided by the tumor proportion score, indicating the percentage of PD-L1^+^ tumor cells (*20*). We stratified patients with NSCLC by PD-L1 expression based on histological assessment of clinical diagnoses by our hospital pathology (table S2). While one tumor (NSCLC-patient 1, green) did not express PD-L1 (0 to 1% PD-L1^+^ tumor cells), most tumors showed intermediate expression (2–49% PD-L1^+^ tumor cells, purple), and one patient (NSCLC-patient 5, red) had high PD-L1 expression (100% PD-L1^+^ tumor cells).

Despite being classified as immune-inflamed, NSCLC-patient 5 (orange) with high PD-L1 expression did not show GrB and CC3 induction following OMTX305 treatment (Fig. 7, G to I), suggesting immune exhaustion or resistance. In contrast, T cells were activated in response to OMTX305 in all other NSCLC samples, with the strongest apoptotic response observed in NSCLC-patient 1 (green), which had no detectable PD-L1 expression. However, it has to be considered that the high CC3 density in this particular sample may have been influenced by a small FAP^+^ area.

In summary, these findings suggest that both patients with NSCLC and HGSOC can respond to OMTX305 therapy. While the limited size of the cohort does not allow for statistical subgroup comparison, exploratory results indicate that, in HGSOC, the baseline immune phenotype may serve as a useful predictor of treatment efficacy. In NSCLC, where immune infiltration was higher, PD-L1 expression may be considered as an additional factor. This observation has to be confirmed in larger patient cohorts.

### OMTX305 drives T cell activation and modulates subset-specific gene expression across HGSOC immune phenotypes

To assess the effect of OMTX305 treatment on T cell and CAF subpopulations from HGSOC patients across different immune phenotypes, we profiled single-cell transcriptomes of control and PBMC cocultured, OMTX305-treated PCTS of four patients with HGSOC (patients 1 (immune-desert), 2 and 3 (immune-excluded), and 9 (immune-inflamed) using the 10x Genomics single-cell RNA-seq platform.

**Fig. 8.**
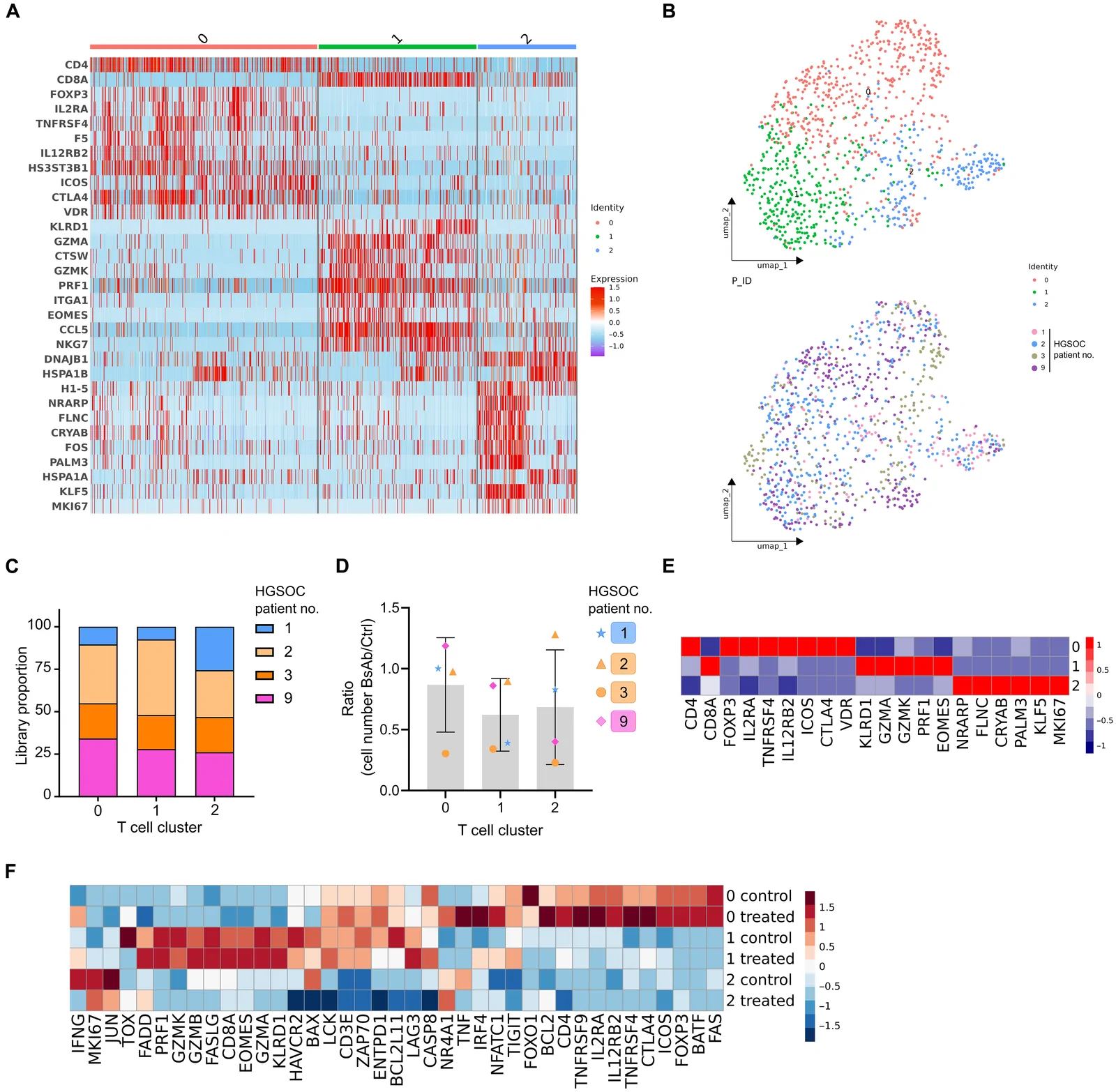
Single-cell RNA-seq of four HGSOC patient PCTS verifies OMTX305-induced T cell activation across HGSOC immune phenotypes. (**A** to **F**) T cell subsets of control and FAP × CD3 BsAb (OMTX305)-treated PCTS of four HGSOC patients (patients 1, 2, 3 and 9) were analyzed by single-cell RNA-seq. (A) Heatmap derived from unsupervised clustering of T cells showing marker genes associated with different T cell clusters. (B) UMAP indicating T cell subclusters colored by subcluster (top) or patient origin (bottom). (C) Library proportions of each patient across T cell subclusters. (D) T cell subcluster ratio of BsAb-treated compared to control PCTS. (E) Heatmap showing gene signatures of T cell subclusters. (F) Gene expression in BsAb-treated and untreated samples in T cell subclusters.

Unsupervised clustering identified three T cell subclusters (clusters 0 to 2) that were conserved across the four patients (Fig. 8, A to C). Consistent with its minimal baseline infiltration (Fig. 5B), the immune-desert HGSOC-patient 1 contributed the fewest T cells (Fig. 8C) to the single-cell RNA cohort. Following OMTX305 treatment, the overall number of T cells was largely preserved in patients with HGSOC (Fig. 8D). All T cell subclusters were reduced by treatment in patient 3, consistent with increased CC3 in T cells observed in mIF stainings (Fig. 6F, HGSOC-patient 3 represented by a black circle).

The largest T cell subcluster, cluster 0, corresponded to CD4^+^ regulatory T cells, defined by the coexpression of *FOXP3*, *IL2RA*, and *CTLA4*, together with activation markers such as *ICOS*, *TNFRSF4*, and *IL12RB2*. Cluster 1 consisted of effector CD8^+^ T cells, characterized by *CD8A*, *KLRD*, and *EOMES*, and enrichment for cytotoxic effector molecules including *GZMA*, *GZMK*, and *PRF1* (Fig. 8E). Cluster 2 comprised a small, highly proliferative T cell population, which was marked by elevated expression of *MKI67*, *FOS*, and *DNAJB1* (Fig. 8, A and E).

OMTX305 induced a coordinated activation program in CD4^+^ T cells (Fig. 8F), as evidenced by the up-regulation of T helper 1–associated cytokines (*IFNG* and *TNF*) and costimulatory receptors (*ICOS*, *TNFRSF4*, and *TNFRSF9*). In parallel, treatment enhanced MYC, E2F, and interleukin-2 signaling pathways, supporting an expansion of CD4^+^ T cells (fig. S9). This activation was accompanied by the induction of immune checkpoints and regulatory feedback molecules, including *CTLA4*, *ENTPD1*, and *NR4A1*, indicating the engagement of compensatory inhibitory pathways. Notably, these were not associated with a pro-apoptotic switch, as *FAS*, *FASLG*, *FADD*, and *CASP8* remained unchanged or reduced following OMTX305 treatment (Fig. 8E).

In CD8^+^ T cells, OMTX305 promoted differentiation toward a cytotoxic effector phenotype, as indicated by enhanced expression of *IFNG*, *PERF1*, and *GZMB*, along with activation-associated transcription factors such as *NFATC1.* Besides, inhibitory receptors (*TIGIT* and *LAG3*) and proapoptotic molecules *FADD* and *CASP8* were up-regulated. By contrast, *FAS* and *FASLG* remained unchanged, and the terminal exhaustion marker *TOX* was down-regulated, which may suggest preservation of effector potential despite checkpoint induction (Fig. 8E).

### OMTX305 induces ECM remodeling and interferon responses in CAFs

**Fig. 9.**
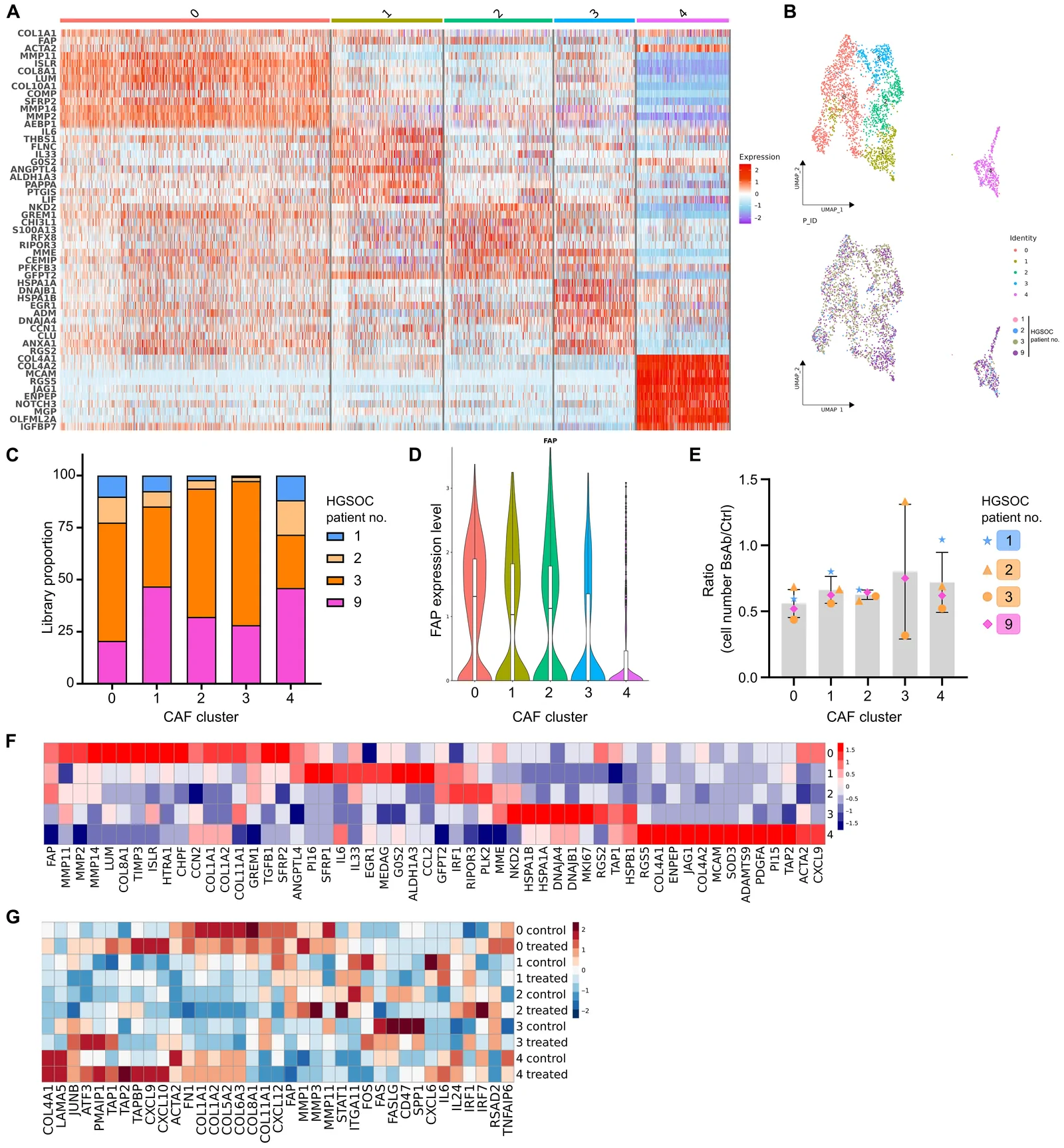
Single-cell RNA-seq of four HGSOC patient PCTS identifies five distinct fibroblast subpopulations and reveals differential responses to FAP × CD3 BsAb (OMTX305) treatment. (**A** to **G**) CAF subsets of control and FAP × CD3 BsAb (OMTX305)-treated PCTS of four HGSOC patients (patients 1, 2, 3 and 9) were analyzed by single-cell RNA-seq. (A) Heatmap derived from unsupervised clustering of CAFs showing marker genes associated with different CAF clusters. (B) UMAP indicating CAF subclusters colored by subcluster (top) or patient origin (bottom). (C) Library proportions of each patient across CAF subclusters. (D) Violin plot showing FAP expression of CAF subclusters. (E) CAF subcluster ratio of BsAb-treated compared to control PCTS. (F) Heatmap showing gene signatures of CAF subclusters. (G) Gene expression in BsAb-treated and untreated samples in CAF subclusters.

Unsupervised clustering of stromal cells revealed 5 distinct CAF subclusters (clusters 0 to 4), present in both untreated and OMTX305-treated samples, each displaying differential FAP expression and variable distribution across patients (Fig. 9, A to D, and fig. S10A). Cluster 0 exhibited the highest FAP expression, followed by clusters 1 and 2 with intermediate levels, while clusters 3 and 4 expressed low FAP levels. The immune-desert HGSOC-patient 1 contributed the lowest number of stromal cells to the single-cell RNA cohort (fig. S10B).

OMTX305 treatment led to a reduction in total stromal cell numbers across all patients. This effect was slightly stronger in FAP^high^ subpopulations. (Fig. 9, D and E).

Among the identified subpopulations, we identified a FAP^high^ myofibroblast cluster (cluster 0) with an elevated expression of extracellular matrix (ECM) genes, an inflammatory and metabolically active IL-6^high^ CAF cluster (cluster 1, FAP^intermediate^), an interferon-response cluster (cluster 2, FAP^intermediate^), a heat shock protein^high^ cluster (cluster 3, FAP^low^), and a pericyte cluster (cluster 4, FAP^low^), similar to CAF subpopulations described in other studies (Fig. 9F) (*21*–*24*).

In response to OMTX305 treatment especially the cluster 0 myofibroblast subset exhibited changes in ECM-related genes, such as a reduced collagen production (*COL1A1*, *COL1A2*, *COL6A3*, and *COL11A1*) and increased expression in matrix metalloproteinases (MMPs) (*MMP1*, *MMP3*, and *MMP11*). Across different subsets, interferon response genes (*IRF1*, *IRF7*, *RSAD2*, and *CXCL9*) and stress-response genes (*ATF3* and *PMAIP1*) were induced by OMTX305 treatment, alongside genes involved in antigen presentation (*TAPBP*, *TAP1*, and *TAP2*) (Fig. 9G and fig. S10C).

In summary, the single-cell RNA-seq analysis of PCTS revealed a reduction in CAF numbers induced by OMTX305 treatment across different immune phenotypes. While FAP^high^ myofibroblasts indicated that OMTX305 treatment induced ECM remodeling, an IFN response was triggered across different CAF clusters.

## DISCUSSION

Because of its broad expression across different tumor entities, targeting of FAP-expressing CAFs is a promising therapeutic approach to modulate the immune suppressive tumor stroma and activate T cells within the TME (*4*, *25*, *26*). In this study, we developed a bispecific, trivalent FAP × CD3 T cell engager and demonstrated significant cytotoxic effects on FAP^+^ CAFs, modulating T cell activity and tumor cell viability in patient-derived model systems of solid tumors, all originating from primary patient material.

In primary tumors, CAFs are a heterogeneous cell population and the majority, but not all, subsets are protumorigenic (*26*). In pancreatic cancer mouse models, the systemic depletion of αSMA-expressing CAFs resulted in enhanced tumor invasiveness (*27*, *28*), whereas the depletion of FAP-expressing CAFs in a similar context reduced tumor malignancy (*28*). In late-stage lung cancer, a CAF subset expressing FAP and αSMA was shown to drive tumor immune exclusion by contributing to a dense ECM (*21*). While the therapeutic targeting of CAFs thus requires careful subset selection to avoid adverse outcomes, FAP is indicated to be a promising target. In this study, OMTX305-induced effects demonstrated FAP dependency across all investigated tumor models. In both 2D and 3D cultures, cell death was induced in FAP^+^ CAFs, but not in FAP^−^ tumor cells. Although FAP expression was induced in NFs in culture, likely due to a phenotypic drift (*14*, *15*), relative FAP expression correlated with the extent of cell death induction within primary CAF and NF pairs. In PCTS, only a subset of CAFs expressed FAP, in accordance with CAF heterogeneity in primary tumors. In mIF stainings, treatment with OMTX305 selectively targeted FAP^+^ areas while sparing αSMA^+^FAP^−^ areas. The absence of specific binding of the OMTX305 anti-FAP scFv sequence in human nontumor tissues supports a low risk of on-target, off-tumor toxicities, thereby minimizing the potential for treatment-related adverse events in vivo.

To elicit cytotoxicity, OMTX305 treatment requires the presence of CD3^+^ T cells. In both CAF spheroids and heterotypic spheroids cocultured with allogeneic PBMCs, OMTX305 treatment enhanced T cell infiltration. In PCTS, coculture with autologous PBMCs did not induce changes in the T cell density. However, the T cell density was significantly decreased by OMTX305 treatment after 3 days. T cell infiltration may have been hindered by the dense and immunosuppressive TME. This may be improved upon a prolonged treatment period, since analysis of CAF subsets by single-cell RNA-seq indicated changes in expression of collagens and MMPs that could contribute to remodeling of the TME. Alternatively, a T cell loss in response to a strong T cell activation may have masked an OMTX305-induced T cell accumulation in PCTS. Neither mIF stainings nor single-cell RNA-seq indicated broad apoptosis induction in T cells after 2 days of treatment, but rather patient-specific effects. Nonetheless, the induction of activation-induced cell death in T cells after 3 days could be associated with the observed increase in IFN-γ or FasL expression in the PCTS system or a lack of costimulatory signaling (*29*, *30*). The benefits of providing T cell costimulatory signals have been shown in bispecific TCE (BiTE)–treated mouse models of solid tumor as well as in a clinical study of a T cell costimulatory 4-1-BB bispecific fusion protein targeting FAP (*31*, *32*), which induced T cell activation and proliferation and increased CD8^+^ T cell infiltration in tumors. Accordingly, the analysis of CAF subsets by single-cell RNA-seq indicated changes in expression of collagens and MMPs, which may remodel the TME and enable T cell infiltration upon a prolonged treatment period.

In the presence of both FAP and T cells, OMTX305 significantly activated T cells, leading to target cell killing in primary CAF-based spheroids. Further, in both HGSOC and NSCLC PCTS, treatment with OMTX305 induced a profound response, conveyed by induction of GrB, Perforin, Granulysin, IFN-γ and FasL. Elevated cytokine concentrations were also observed in the absence of cocultured PBMCs, indicating that OMTX305-driven T cell activation is predominantly mediated by tissue-resident T cells in PCTS. Notably, coculture with PBMCs further enhanced T cell activation in four patients, indicating a potential contribution of PBMC-derived T cells. However, this observation requires validation in a larger cohort. Despite the reduced T cell density observed in PCTS in response to OMTX305 treatment, increased GrB levels were maintained within tumor slices during the 3-day culture period. Nevertheless, OMTX305 responses varied between PCTS from different patients and were at least partially correlated to the functional state of T cells. At the transcriptional level, OMTX305 induced a coordinated activation program in CD4^+^ T cells and promoted cytotoxic differentiation in CD8^+^ T cells, accompanied by up-regulation of costimulatory and effector pathways across all HGSOC immune phenotypes. While HGSOC samples showed a range of immune phenotypes, all NSCLC samples were immune-inflamed. Although cutoffs for immune exclusion may vary between entities, our cohort thus reflects the immunogenic profiles of both entities studied, with HGSOC generally being considered less immunogenic than NSCLC, with a lower number of immune infiltrates and a reduced immunotherapy response (*18*, *33*–*35*).

In ovarian cancer, T cell–based immune phenotyping is prognostic and has been repeatedly proposed as a biomarker (*33*, *36*). Accordingly, despite the limited patient cohort, within our study, immune-infiltrated HGSOC tumors showed among the strongest response to OMTX305 treatment. In contrast, the immune-desert tumor, although represented only by a single case, maintained its cold phenotype. Intriguingly, T cell activation and cell death induction in FAP^+^ CAFs and tumor cells were observed in immune-excluded patients—highlighting that this patient subgroup, in which other immunotherapies such as immune checkpoint blockade (ICB) fail to induce a response (*16*, *17*, *33*), may benefit from OMTX305 treatment.

Besides a lack of T cells within the TME, T cell exhaustion represents a challenge in the efficacy of T cell–based immunotherapies (*37*, *38*). Especially in NSCLC, PD-L1 expression as a measure of T cell exhaustion supports patient stratification to predict clinical outcomes (*39*, *40*). Preclinical studies have demonstrated that combining TCEs with PD-1 blockade can enhance the antitumor activity (*41*, *42*). Consistent with this, in PCTS experiments, the patient with NSCLC with the highest PD-L1 expression in tumor cells failed to respond to OMTX305 treatment (Fig. 7). While these ex vivo results have to be verified in larger cohorts, they support the rationale for combining FAP-targeting TCEs with ICB blockade, a strategy currently under clinical investigation (*31*, *43*).

In both heterotypic spheroids and PCTS, OMTX305 induced apoptosis in tumor cells adjacent to FAP^+^ areas. These bystander killing effects in response to CAR-T and BsAb therapies have been previously attributed to Fas-FasL signaling (*44*–*46*) and soluble pro-inflammatory factors (*47*, *48*). Consistently, OMTX305 treatment induced FasL and IFN-γ expression, suggesting their contribution to the observed bystander tumor cell death. IFN-γ–mediated bystander effects have been observed to span distances of up to 250 to 800 μm (*48*, *49*). In line with this, apoptotic tumor cells were located between 10 and 100 μm from the FAP^+^ area in our experiments. Notably, similar bystander killing of FAP-targeting strategies have been reported in mouse models of pancreatic cancer and glioblastoma treated with FAP-specific CAR T cells (*47*, *50*). More broadly, the potential of FAP-targeting in solid tumors has been demonstrated across multiple modalities, including cytolysin-mediated secondary killing induced by the anti-FAP ADC OMTX705 (*51*, *52*), FAP-targeted radionuclide delivery (LNC1004) (*53*), and FAP-dependent localized immune activation (RO7122290; BI 754091) (*43*, *54*). These approaches are being evaluated in clinical trials across multiple solid tumor entities including NSCLC, pancreatic adenocarcinoma, and colorectal cancer.

In this study, we demonstrated the ability of the FAP-targeting OMTX305 to target and remodel the TME in PCTS, highlighting the versatile potential of PCTS as a platform for translational immunotherapeutical research. PCTS offer a low-risk, low-cost approach to investigate responses to nonapproved experimental treatments. Further, they enable repeated sampling to track treatment responses over time, as well as direct comparisons of untreated and various drug-treated tumor tissues derived from the same patient sample. Using PCTS, initial results predicting a tumor’s response to treatment within its natural TME can be obtained within a week, in contrast to organoid- or cell-based models, which require more time for establishment (*55*). However, there are also some limitations of our study. First, the culture period of PCTS is restricted. Thus, long-term changes in ECM composition elicited by a reduced FAP^+^ fibroblast number and subsequent effects on the tumor cannot be studied. Moving forward, the potential of PCTS to guide patient selection has to be validated in larger patient cohorts.

In conclusion, our findings support a continued clinical development of OMTX305. Further, investigating combinational strategies should be considered, either with ICB to target immune exhaustion or with T cell stimulatory molecules to expand the pool of available tumor-associated T cells.

## MATERIALS AND METHODS

### FAP × CD3 TCEs

Construction, production and in vitro testing of bispecific TCEs targeting FAP and CD3 were performed using established protocols (*12*, *56*). The eIg and Fab-eIg FAP × CD3 TCE were built using a humanized version (hu36) of the anti-FAP antibody MO36 (*57*) and a humanized version of the anti-CD3 antibody UCHT1. Both TCEs further comprised a heterodimerizing knobs-into-holes Fcγ1 and were silenced for Fc-mediated effector functions by introducing mutations into the lower hinge and the CH2 domain (*12*).

### Study design

The aim of this study was to track the response of complex patient-derived models of solid tumors to OMTX305 treatment. The sample size for 2D and 3D CAF experiments (*n* = 3) was chosen to provide a reasonable level of statistical power for detecting meaningful differences. In the figure legends, biological replicates are indicated by *n*, together with the applied statistical analysis. The aim of the CAF experiments was to measure T cell infiltration, T cell activation, and cytotoxicity induction by viability assays, live fluorescence microscopy, and stainings by IHC and mIF. The choice of time points and drug concentrations for these analyses was based on initial time course and dose-response experiments. Human tumor and blood samples for PCTS experiments were received from patients with HGSOC or NSCLC undergoing debulking surgery. The sample size for PCTS experiments (*n* = 15) was based on previous experience using the PCTS platform (*58*). In these PCTS experiments, the primary outcome was mIF stainings to determine T cell infiltration and activation, as well as cell death induction in different tumor slice regions and different cell types in response to OMTX305 treatment. The data were supported by viability analysis, cytokine determination, single-cell RNA-seq, and immunofluorescence imaging. The choice of analyzed time points was based on preceding cell line experiments. The BsAb format and drug concentration was chosen on the basis of in vitro studies with HT1080-FAP cells. To account for intratumoral heterogeneity, slices were numbered during the slicing process and distributed evenly among different conditions. Tumors that were unsuitable for PCTS slicing due to their size or structure were excluded from experiments. Studies were not blinded. For mIF image analysis, whole cross-sections of PCTS were analyzed, excluding necrotic areas. For cell-type determination and biomarker expression, the same algorithm was used for all slices of one patient. All studies including human samples were approved by the ethics committee of the University Hospital Tübingen, Germany (810/2023BO2, 397/2016BO1, and 15-/2011BO2). Informed consent was obtained from all subjects whose biological materials were involved in this study.

### Cells

The fibrosarcoma cell line HT1080 WT and the HT1080-FAP derivative thereof stably expressing human FAP were provided by W. Rettig (Boehringer Ingelheim Pharma) (*59*). The NSCLC cell lines H1437 (#CRL-5872) and A549 (#CCL-185) were acquired from American Type Culture Collection (Manassas, Virginia, USA). The fibroblasts used in this study were isolated from treatment-naïve lung tissues of NSCLC patients obtained after surgical resection at the Robert Bosch Hospital (Stuttgart, Germany). Matched CAFs and NFs were isolated from tumor tissue and adjacent noncancerous tissue. For this study, three primary pairs of CAF and NF were used (Primary CAF-001, Primary CAF-002, Primary CAF-003, and their corresponding NFs Primary NF-001, Primary NF-002, Primary NF-003). In addition, two immortalized pairs of CAF and NF were included (IKP-CAF-001 and IKP-NF-001 derived from patient tissue with NSCLC adenocarcinoma; IKP-CAF-002 and IKP-NF-002 derived from patient tissue with NSCLC adeno-squamous carcinoma). The isolation process is as described in (*60*). Briefly, NSCLC tissue was minced and digested enzymatically. The resulting cell solution was passed through a 70-μm filter before separating the cell suspension into CD326^+^ tumor cells and CD326^−^ fibroblasts by magnetic-activated cell sorting. The CD326^−^ cells were cultured as primary NSCLC CAFs. For immortalized CAF/NF pairs, immortalization was conducted using a lentivirus coexpressing human telomerase reverse transcriptase (TERT) and GFP (Lenti-hTERT-eGFP; catalog no. LG508, BioGenova) as described in (*13*).

For in vitro coculture experiments, allogeneic PBMCs were obtained from healthy donor blood from the DRK Blutspendedienst Baden-Württemberg-Hessen. Experiments with allogeneic PBMCs were repeated three times, each time with PBMCs from a different healthy donor. Autologous PBMCs for PCTS coculture were derived from patient blood. PBMCs were isolated from blood by density gradient centrifugation following standard protocols. If cryopreserved, PBMCs were thawed the day before the start of the experiment and cultured overnight at 37°C in a suspension culture in RPMI 1640 medium supplemented with GlutaMAX, 10% fetal bovine serum (FBS), and 1% penicillin-streptomyin (P/S).

### Cell culture

Both NSCLC cell lines (H1437 and A549) were cultured in RPMI 1640 medium supplemented with GlutaMAX (catalog no. 61870036, Gibco, Grand Island, NY, USA), 10% FBS (#S0615, Sigma-Aldrich, St. Louis, Missouri, USA), and 1% P/S (#15140163, Gibco). Fibroblasts were cultured in RPMI 1640 medium supplemented with GlutaMAX, 10% FBS, 1% P/S, and 0.6× MEM nonessential amino acids (NEAA, #P08-32100, PAN-Biotech, Aidenbach, Germany).

### Preparation of ultralow attachment plates

Ultralow attachment plates were prepared by coating U-bottom 96-well plates with poly-2-hyproxyethyl methacrylate (poly-HEMA; #P3932, Sigma-Aldrich, St. Louis, Missouri, USA). To each well, 150 μl of poly-HEMA (20 mg/ml) in 95% ethanol was added. Plates were incubated shaking at 350 rpm for 4 hours. Then, 75 μl of poly-HEMA was removed, and plates were left to dry in a laminar hood overnight for complete evaporation of the ethanol. Once they were dried, plates were stored at 4°C until use.

### Spheroid culture

Cell suspensions of IKP-CAF-001 or H1437 were seeded at 3000 cells per well in 50-μl medium in poly-HEMA–coated ultralow attachment plates. To generate heterotypic spheroids, IKP-CAF-001 and H1437 cells were mixed in a 3:1 ratio, for a total cell number of 3000 cells per well. Spheroids were cultured in fibroblast medium (see cell culture). Plates were centrifuged at 300*g* for 3 min and incubated at 37°C in a humidified incubator overnight.

### Drug treatment of cells and spheroids

After an overnight incubation, cells or spheroids were cocultured with allogeneic PBMCs at a 10:1 (effector:target) cell ratio and treated with varying concentrations of FAP × CD3 BsAb (OMTX305).

### MTT assay

For the MTT cell viability assay, 2000 cells per well were seeded in 96-well plates. After overnight attachment, allogeneic PBMCs were added at a 10:1 (effector:target) cell ratio, and cells were exposed to varying concentrations of FAP × CD3 BsAb (OMTX305). Each experimental condition was assessed in technical triplicates. After incubation for 72 hours, the supernatant was removed and 100 μl of a MTT solution (0.5 mg/ml) in dimethyl sulfoxide (DMSO) was added to each well. Following a 2-hour incubation period, the MTT solution was discarded, and the formed formazan crystals were dissolved in 100 μl of DMSO. The optical density was measured at 562 nm using the VICTOR Nivo Plate Reader (Revvity, Waltham, Massachusetts, USA).

### LDH assay

For the lactate dehydrogenase (LDH) release assay, spheroids were cocultured with PBMCs and treated with increasing concentrations of FAP × CD3 BsAb (OMTX305). A complete medium control without cells was included to determine LDH background activity in the serum. To assess cytotoxicity, maximum LDH activity controls (achieved by complete cell lysis), and untreated spontaneous LDH activity controls were included in the assay.

Following spheroid formation overnight, PBMCs were added at a 1:10 target:effector cell ratio, along with varying concentrations of FAP × CD3 BsAb (OMTX305). Each experimental condition was measured in technical triplicates. After incubation for 72 hours, the LDH release assay was performed using the CyQUANT LDH Cytotoxicity Assay (#C20300, Invitrogen, Carlsbad, California, USA) according to the manufacturer’s protocol. Absorbance was measured at 490 and 680 nm. Cytotoxicity was calculated using the following formula

$$\begin{array}{c} \%\text{Cytotoxicity}=\\ \left[\frac{\text{compound treated}\;\text{LDH}\;\text{activity}-\text{spontaneous}\;\text{LDH}\;\text{activity}}{\text{maximum}\;\text{LDH}\;\text{activity}-\text{spontaneous}\;\text{LDH}\;\text{activity}}\right] \end{array}$$

### Real-time qPCR

RNA was isolated using the QIAGEN RNeasy Mini Kit (#74104, QIAGEN, Venlo, Netherlands) and reverse transcribed into cDNA using M-MLV RT (M1701, Promega, Madison, Wisconsin, USA). TaqMan assays (Applied Biosystems, Foster City, CA, USA) for FAP (Assay ID: Hs00990791_m1) and TATA-box binding protein (TBP, Assay ID: Hs00427620_m1) were run with the QuantStudio 5 quantitative polymerase chain reaction (qPCR) System (ABI Applied Biosystems Waltham, MA, USA).

### Primary human tumors

Fresh NSCLC or HGSOC tumor tissue was obtained from debulking surgery at the Robert Bosch Hospital (Stuttgart, Germany). Tumor classification was confirmed through histopathological examination by experienced pathologists. Detailed patient information is provided in tables S1 and S2.

### Tumor slice preparation

PCTS were prepared following previously established protocols (*61*). Tumor tissue was sectioned into 1 to 1.5 cm^3^ cubes and fixed on a magnetic specimen holder of a Leica VT1200S automated vibrating blade microtome (VT1200S, Leica, Wetzlar, Germany) using cyanoacrylate adhesive. The PCTS of 250-μm thickness were prepared. Depending on the consistency and condition of the tumor tissue, 15 to 25 PCTS were obtained from each tumor cube. Tumor tissue harvested for analysis on the day of slicing was defined as d0.

### Tumor slice coculture and treatment

PCTS were cultured at an air-liquid interface on a filter support (Millicell cell culture inserts, PTFE, pore size of 0.4 μm, PIGM0RG50, Merck Millipore, Burlington, MA, USA) in six-well plates. A total of 1.5-ml medium was added below the filter. One drop of medium was added on top of each PCTS to prevent their drying. Slices were cultured in RPMI 1640 with GlutaMAX (61870036, Gibco, Grand Island, NY, USA), supplemented with 5% FBS (Superior, S0615, Sigma-Aldrich, St. Louis, Missouri, USA), penicillin (100 U/ml), and streptomycin (100 μg/ml). For OMTX305 treatment, 100 nM OMTX305 was added to the culture medium. For coculture experiments, 8 × 10^5^ PBMCs were resuspended in 250-μl medium, with or without 100 nM OMTX305, and added on top of the slices. PCTSs were cocultured and treated for up to 3 days and then harvested for ATP assay or formalin-fixation and paraffin-embedding (FFPE) for subsequent stainings. Depending on tissue availability, additional PCTS were harvested on days 1 and 2. For single-cell RNA-seq, PCTS were harvested on day 2. Medium was exchanged daily, with half of the medium collected and replaced with fresh medium. The collected medium was snap-frozen and stored at −80°C for subsequent cytokine analysis.

### ATP assay

To determine ATP levels of tumor tissue (d0) or tumor slices, three tumor slices per condition were snap-frozen in Lysing Matrix D tubes (#9554.1, MP Biomedicals, Santa Ana, CA, USA) and stored at −80°C until processing. Each sample was then homogenized by adding 1000 μl of 2 mM EDTA in 70% EtOH (pH 10.9). Samples were lysed at 4°C using a homogenizer (FastPrep-24, MP Biomedicals) for 30 s at 6 m/s, repeated twice, and then centrifuged for 10 min at 9300 rcf. The resulting supernatant was diluted 1:10 in 100 mM tris and 4 mM EDTA (pH 7.75) and used for ATP quantification via the ATP Bioluminescence Assay Kit CLS II (#11699695001, Roche, Basel, Switzerland) following the manufacturer’s protocol. ATP levels were measured in technical triplicates. The precipitate was used to measure protein levels for normalization. After drying at 37°C overnight, the precipitate was resuspended in 200 μl of 5 N NaOH and incubated at 37°C shaking at 1000 rpm for 30 min. Then, the NaOH was diluted to 1 N with Millipore-H_2_O. Samples were lysed in a homogenizer for 30 s at 6 m/s. Protein concentration was determined using the Pierce BCA Protein Assay Kit (#23227, Thermo Fisher Scientific, Waltham, Massachusetts, USA) according to the manufacturer’s instructions. Both the BSA standard and the samples were measured in technical triplicates. ATP levels were normalized to protein content.

### Formalin-fixation and paraffin-embedding

For FFPE, cells or spheroids were harvested and resuspended in HistoGel (catalog no. HG-4000-012, Epredia, Thermo Fisher Scientific, Waltham, MA, USA). The HistoGel drops were then fixed in 4% formaldehyde. PCTS were paraffin-embedded in a vertical orientation as described by Davies *et al.* (*55*). Dehydration and paraffin embedding were performed at the pathology department of the Robert Bosch Hospital (Stuttgart, Germany).

### Immunohistochemistry

FFPE samples were sectioned at 3 μm thickness using a rotary microtome (RM2255, Leica, Wetzlar, Germany) and dried overnight at 56°C. Immunohistochemical (IHC) staining was carried out as described previously using standard protocols. Briefly, sections were deparaffinized in Neoclear (catalog no. 109843, Sigma-Aldrich, St. Louis, Missouri, USA), rehydrated through a graded ethanol series and stained using the Dako Envision Kit (#K5007, Agilent Technologies, Glostrup, Denmark) according to the manufacturer’s instructions. Heat-induced epitope retrieval was performed in a steam heater for 30 min using either a pH 6 citric acid buffer (S2369, Agilent Technologies) or a pH 9 tris/EDTA buffer (S2367, Agilent Technologies). The following antibodies were used: cleaved caspase-3 (Asp^175^, 1:75; #9661, Cell Signaling Technology, Danvers, MA, USA), EpCAM (Ber-EP4, 1:100; M0804, Agilent Technologies, Glostrup, Denmark), and FAP (EPR20021, 1:100; ab207178, Abcam, Cambridge, UK). The Dako REAL Envision/HRP Rabbit/Mouse antibody provided within the Dako Envision Kit was applied as a secondary antibody. Antibodies were detected with 3,3′-diaminobenzidine, and sections were counterstained with hematoxylin. Stained sections were imaged using an Olympus VS120 slide scanner (Olympus Life Science, Waltham, MA, USA).

### Multiplex immunofluorescence staining

Seven-color mIF staining was performed using the tyramide signal amplification-based OPAL multiplexing method which allows simultaneous detection of six antibodies plus 4′,6-diamidino-2-phenylindole (DAPI). FFPE tissues were cut into 3-μm sections using a rotary microtome (Leica R2255, Leica Biosystems, Nussloch, Germany). Sections were deparaffinized, rehydrated, and subjected to heat-induced epitope retrieval before incubation with primary and secondary antibodies. Antibodies were visualized with fluorescent tyramides of the Opal 6-Plex Manual Detection Kit (NEL861001KT, Akoya Biosciences, Marlborough, MA, USA). The epitope retrieval and staining procedures were repeated sequentially for different primary antibody and fluorescent tyramide combinations. Panels consisting of the following antibodies were optimized: αSMA (1A4, 1:100; ab7817, Abcam, Cambridge, UK), cleaved caspase-3 (Asp175, 1:75; #9661, Cell Signaling Technology, Danvers, MA, USA), CD3 (MRQ-39, 1:100; #103R, Cell Marque, Rocklin, CA, USA), FAP (EPR20021, 1:100; ab207178, Abcam, Cambridge, UK), GFP (D5.1, 1:100; #2956P, Cell Signaling Technology), Granzyme B (D6E9W, 1:100; #46890, Cell Signaling Technology), and pan-Keratin (AE1/AE3, #67306, Cell Signaling Technology). The Opal Polymer HRP Ms+Rb antibody provided within the Opal 6-Plex Manual Detection Kit was applied as a secondary antibody. Nuclei were stained with DAPI. Stained sections were mounted with ProLong Diamond Antifade Mountant (P36961, Thermo Fischer Scientific, Waltham, MA, USA) and imaged using the PhenoImager Fusion slide scanner (Akoya Biosciences).

### Multiplex immunofluorescence staining analysis

Following mIF staining, spectral unmixing of whole spheroid or PCTS cross sections was performed in inForm 3.1.0 (Akoya Biosciences, Marlborough, MA, USA) using tissue-specific autofluorescence controls. Unmixed images were analyzed using QuPath version 4.4.0 (*62*). Whole tissue slices were annotated, excluding necrotic areas. Nuclei were detected with the StarDist extension. FAP^+^ and FAP^−^ areas were determined using a pixel classifier for the FAP channel. Tumor and stroma regions were determined using a pixel classifier for the pan-CK channel. Object classifiers were trained to detect cell types or functional markers. The same algorithm was used for all spheroids of one experiment or all tissue slices of one patient. Immune phenotypes were determined from the d0 tissue using a pixel classifier for the tumor and stroma. The percentage or density of cells (cells per mm^2^) positive for a marker or a combination of markers was calculated. The images were visually reviewed to ensure reliable phenotyping.

### Spatial analysis

Distances between apoptotic (CC3^+^) or nonapoptotic (CC3^−^) tumor cells (pan-CK^+^) and the FAP^+^ region were determined with the “Distance to annotations 2D” function in QuPath. Mean distances were calculated. A quotient of the distance of CC3^+^ and CC3^−^ tumor cells was determined, indicating the relative distance of apoptotic tumor cells to the FAP^+^ region.

### Cytokine analysis

The cytokine levels for Granzyme B, Perforin, Granulysin, Granzyme A, FasL, and IFN-γ were analyzed in the PCTS culture medium by flow cytometry using the LegendPlex Human CD8/NK Panel (13-plex) (#741187, BioLegend, San Diego, CA, USA) according to the manufacturer’s instructions.

### T cell tracking

Autologous T cells were isolated from patient PBMCs using the EasySep Human T Cell Isolation Kit (#17951, STEMCELL Technologies, Vancouver, Canada) according to manufacturer’s instructions and stained with 15 μM CellTracker Orange CMRA (C34551, Thermo Fisher Scientific, Waltham, MA, USA) for 30 min at 37°C. For coculture, 4 × 10^5^ T cells were added on top of PCTS as described for PBMCs. After coculture and treatment, PCTS nuclei were stained with DRAQ5 (DR50050, Biostatus Ltd., Leicestershire, UK) 1:1000 in medium for 30 min before fixation in 4% formaldehyde overnight. The next day, tissue slices were dehydrated and rehydrated in a graded ethanol series and then cleared in Rapiclear 1.49 (#RC149001, Sunjin Lab Co., Hsinchu City, Taiwan) in chambered coverslips at room temperature overnight. Cleared PCTS were imaged by capturing z-stacks with a Leica TCS SP8 confocal laser scanning microscope (Leica, Wetzlar, Germany) and visualized with the Leica Application Suite X 3.5.7.

### Single-cell RNA-seq

After culture and treatment for 2 days, PCTS were snapfrozen and stored at −80°C until processing for single-cell RNA-seq. Tissue collected on d0 or PCTS were fixed and dissociated using the Chromium Next GEM Single Cell Fixed RNA Sample Preparation Kit (1000414, 10x Genomics, Pleasanton, CA, USA). Single-cell RNA-seq was performed using the 10x Genomics Fixed RNA workflow following the manufacturer’s instructions (User Guide CG000527 Rev. D). In brief, the fixed and permeabilized cells or nuclei were incubated with RNA hybridization probes. Following probe hybridization, the samples were loaded onto a 10x Genomics Chromium Next GEM Chip Q, where they were partitioned forming gel bead-in-emulsions (GEMs). Within the GEMs, individual cells or nuclei underwent lysis, following reverse transcription generating barcoded cDNA by incorporating unique cellular barcodes and unique molecular identifiers (UMIs). The barcoded cDNA was then recovered from the GEMs and subjected to a preamplification. Subsequently, libraries were constructed through enzymatic fragmentation, end repair, adapter ligation, and a final PCR step to add sample-specific indices for sequencing. The completed libraries underwent quality control via Bioanalyzer and qPCR, before being pooled and loaded onto an Illumina NovaSeq X plus. Raw sequencing data was then preprocessed using the 10x Genomics Cell Ranger pipeline (ver. 9) for alignment, barcode and UMI correction, and gene expression quantification. Reads were aligned to the GRCh38 human reference genome using STAR within Cell Ranger software. Data processing was performed using the Seurat R package (v4.4.0) (*63*). The resulting count matrices by Cell Ranger were normalized via the LogNormalize method implemented in Seurat. To ensure data quality, transcriptomic doublets were identified and excluded using DoubletFinder (*64*). Batch effect correction was implemented through canonical correlation analysis, followed by dimensionality reduction via principal components analysis. Cell populations were next defined through an unsupervised clustering method using the original Louvain algorithm via “FindClusters” function within the Seurat package. Initial cell-type identities were assigned using SingleR and subsequently refined by cross-referencing canonical markers with the PanglaoDB database (*65*). For downstream analysis, the fibroblast population and the T cell population were isolated for differential gene expression, gene set enrichment analysis, and gene set variation analysis. Visualizations were produced using ggplot2, pheatmap, and EnhancedVolcano.

### Statistical analysis

Statistical analysis was performed using GraphPad Prism 10 (GraphPad Software, Boston, MA, USA). For spheroid experiments, two conditions from three biological replicates with different allogeneic PBMC donors were compared. The statistical significance between the two groups was calculated using two-tailed ratio paired *t* tests. For PCTS experiments, the response of PCTS derived from different patients was analyzed. Only two conditions were compared at a time using a two-tailed Wilcoxon matched-pairs signed-rank test. Each specific test is mentioned in the corresponding figure legend. For all statistical tests, significance was defined as *P* < 0.05 with *P ≤ 0.05, **P ≤ 0.01, and ***P ≤ 0.001. Data are presented as means ± SD.
