# Supplementary material for: The FAP × CD3 bispecific antibody OMTX305 induces T cell–mediated antitumor effects in patient-derived ex vivo models of solid tumors
Source: Sci Adv. 2026 Sep 11;12(37):eaee2124. doi: 10.1126/sciadv.aee2124 (PMC13564906; doi:10.1126/sciadv.aee2124)
Supplement: Supplementary file 1 [file sciadv.aee2124_sm.pdf]

Science Advances
AAAS

Supplementary Materials for

The FAP × CD3 bispecific antibody OMTX305 induces T cell–mediated antitumor effects in patient-derived ex vivo models of solid tumors

Julia Thiel *et al.*

Corresponding author: Meng Dong, meng.dong@ikp-stuttgart.de

Sci. Adv. **12**, eaee2124 (2026)
DOI: 10.1126/sciadv.aee2124

This PDF file includes:

Figs. S1 to S10
Tables S1 to S4

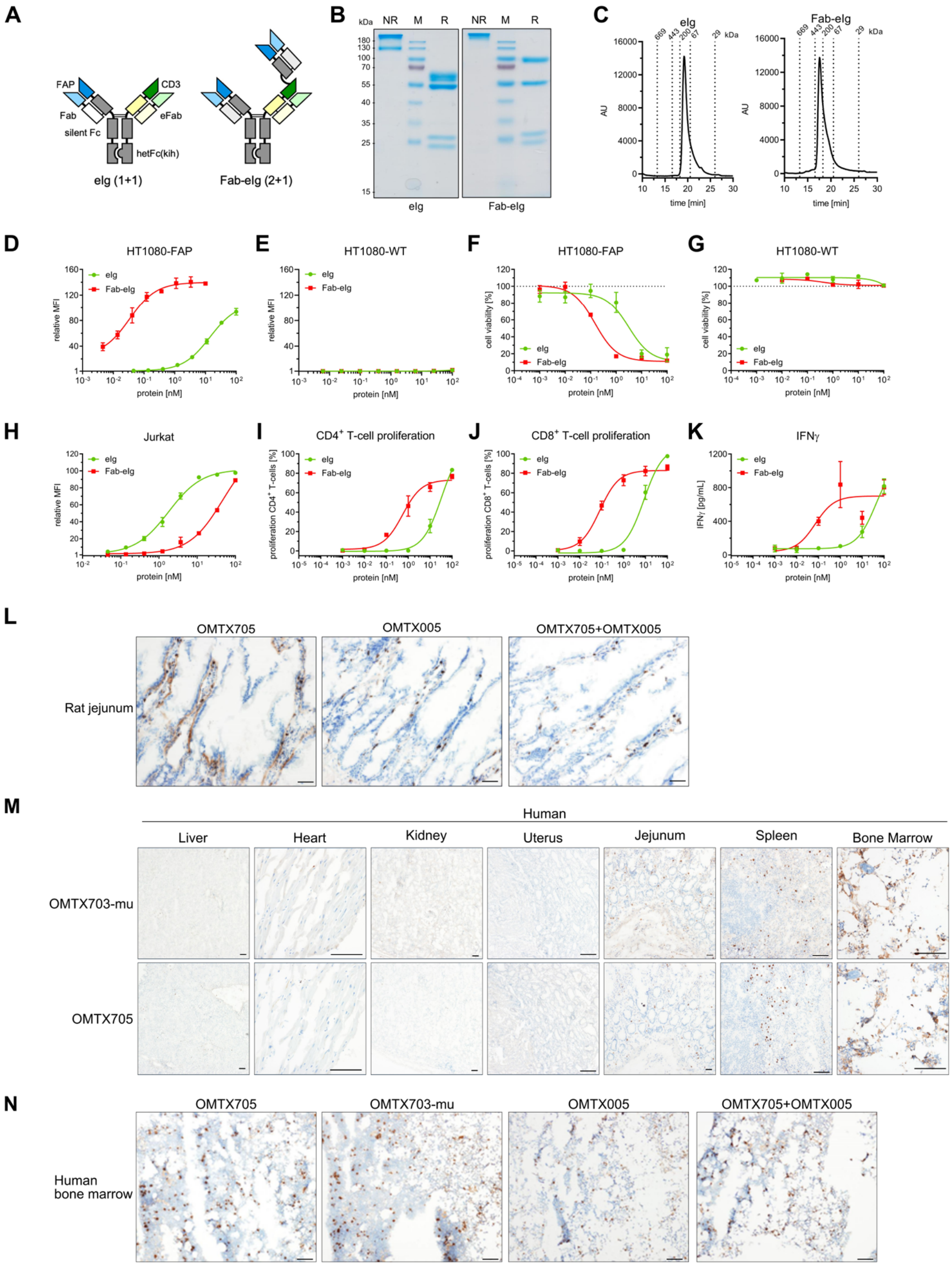

Fig. S1: CD3xFAP T cell engager. (**A**) Composition of the 1+1 eIg and 2+1 Fab-eIg TCE. (**B**) SDS-PAGE analysis of purified TCEs under non-reducing and reducing conditions. (**C**) Size-exclusion chromatography of purified TCEs. (**D-E**) Binding to HT1080-FAP cells and HT1080 wt cells analyzed by flow cytometry. (**F-G**) TCE-mediated killing of HT1080-FAP cells and HT1080 wt cells in the presence of human PBMCs at an E:T ratio of 10. (**H**) Binding to CD3$^+$ Jurkat cells analyzed by flow cytometry. (**I-J**) Activation of proliferation of CD4$^+$ and CD8$^+$ T cells in the presence of TCE and HT1080-FAP cells. (**K**) Induction of IFN-γ release from human T cells after 3 days of incubation of HT1080-FAP cells with PBMCs and TCEs. **(L-N)** Rat or human tissue sections were stained with the FAP-ADC OMTX705 (anti-FAP IgG1 OMTX005+cytolysin TAM558) (10 µg/ml) or a murine negative control OMTX703-mu (10 µg/ml). A polyclonal antibody against the payload TAM558 was used as a secondary antibody. Scale bar, 50 µm. **(L)** Staining of rat jejunum. Competition staining with OMTX005 (100 µg/ml) was performed

to verify staining specificity. **(M)** Staining of human tissue sections of healthy organs with OMTX703-mu as negative control and OMTX705 as target. **(N)** Staining of human bone marrow. Competition staining with OMTX005 (100 µg/ml) was performed to verify non-specificity of the staining. The positive signals of OMTX705 are mainly caused by non-specific binding.

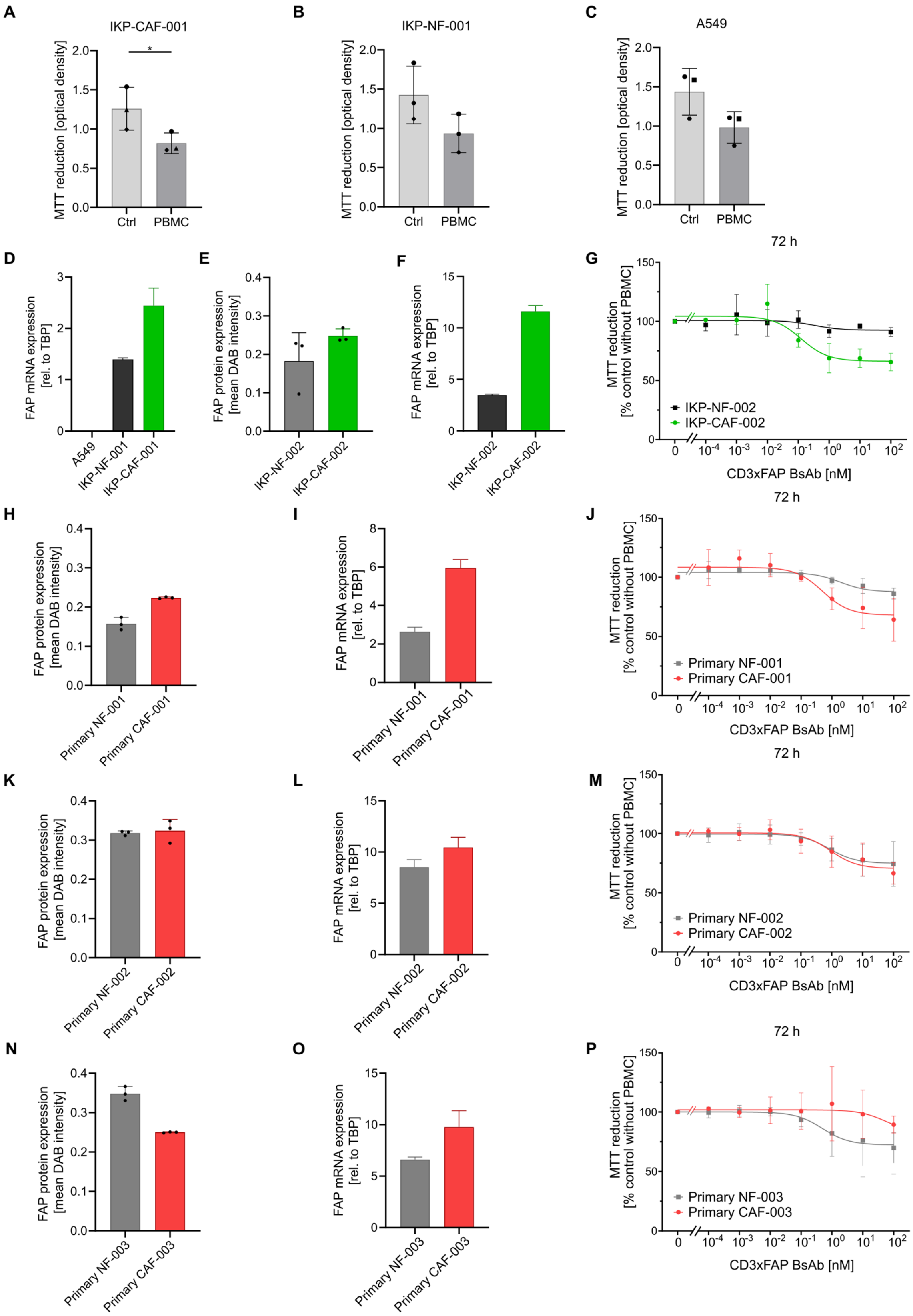

Fig. S2: FAP expression influences CD3xFAP BsAb (OMTX305) treatment responses of patient-derived matched CAF and NF. MTT assay showing viability of **(A)** IKP-CAF-001, **(B)** IKP-NF-001, or **(C)** A549 cells with or without PBMC co-culture (n=3, mean ± SD) (Two-tailed ratio paired t test;

* p ≤ 0.05). (**D-G**) Immortalized CAFs (IKP-CAF-001, IKP-CAF-002) and NF (IKP-NF-001, IKP-NF-002) were derived from NSCLC tumor tissue and adjacent normal lung tissue. (**D**) FAP mRNA expression in IKP-CAF/NF-001 as assessed by qPCR (n=1, mean ± SD of technical triplicates). (**E**) FAP protein expression in IKP-CAF/NF-002 quantified from a FAP IHC staining (Mean ± SD of three areas with >1000 cells/area). (**F**) FAP mRNA expression in IKP-CAF/NF-002 as explained in (D). (**G**) MTT assay showing relative viability of IKP-CAF-002 or IKP-NF-002 co-cultured with PBMCs and CD3xFAP BsAb (OMTX305)-treated for 72 h (n=3, mean ± SD). (**H-P**) Primary CAF/NF (Primary CAF/NF-001; Primary CAF/NF-002; Primary CAF/NF-003) were isolated from NSCLC tumor tissue or adjacent normal lung tissue. (**H**) FAP protein expression in Primary CAF/NF-001 as explained in (E). (**I**) FAP mRNA expression in Primary CAF/NF-001 as explained in (D). (**J**) MTT assay showing relative viability of Primary-CAF/NF-001 as explained in (G). (**K**) FAP protein expression in Primary CAF/NF-002 as explained in (E). (**L**) FAP mRNA expression in Primary CAF/NF-002 as explained in (D). (**M**) MTT assay showing relative viability of Primary-CAF/NF-002 as explained in (G). (**N**) FAP protein expression in Primary CAF/NF-003 as explained in (E). (**O**) FAP mRNA expression in Primary CAF/NF-003 as explained in (D). (**P**) MTT assay showing relative viability of Primary-CAF/NF-003 as explained in (G).

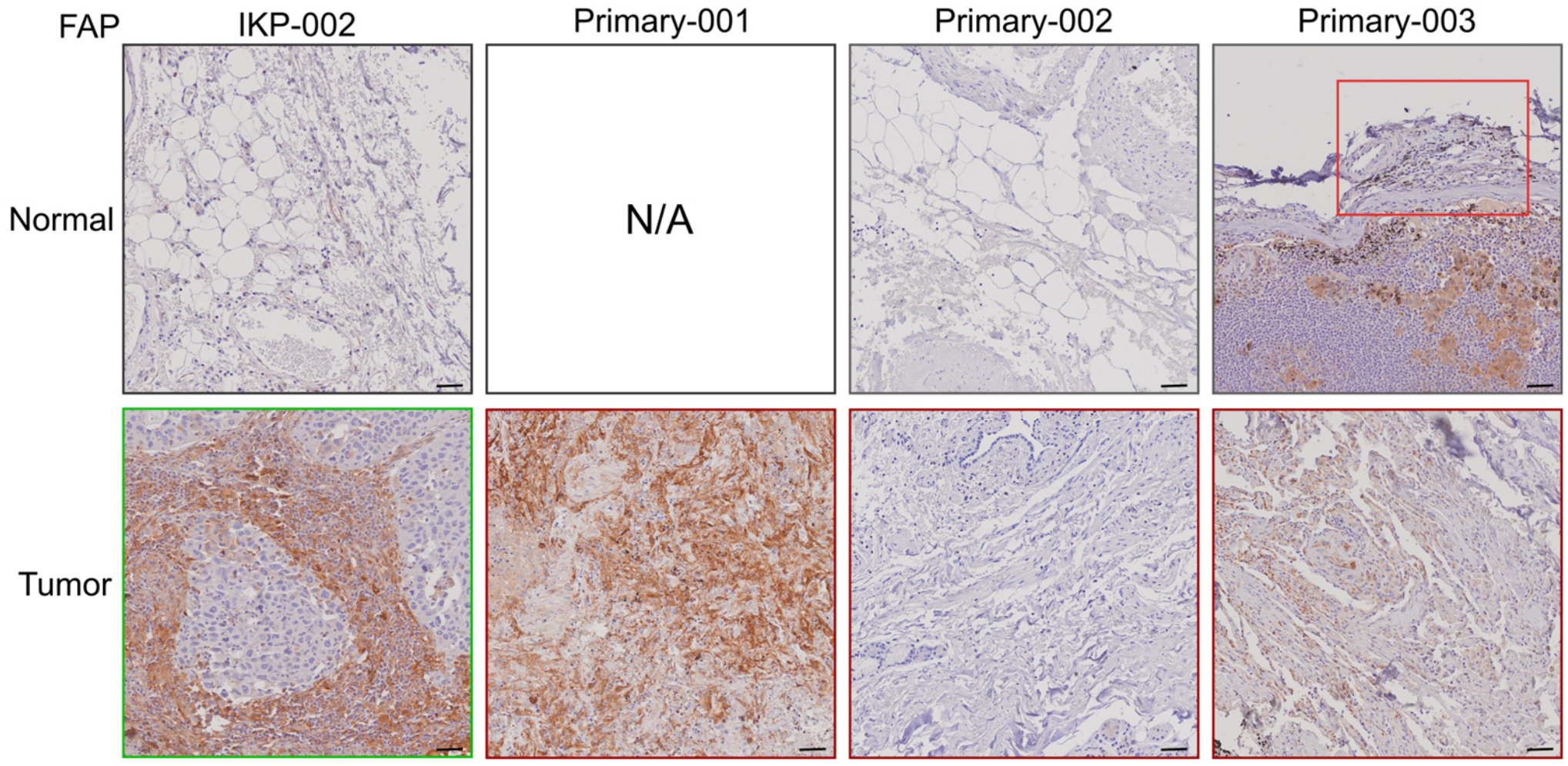

Fig. S3. FAP expression in original tissue of patient-derived matched CAF and NF. FAP expression in NSCLC tumor tissue and adjacent normal lung tissue of matched CAF/NF shown by IHC staining. The red box shows the normal lung tissue of Primary-003, adjacent to a lymph node. Scale bar, 50 μm.

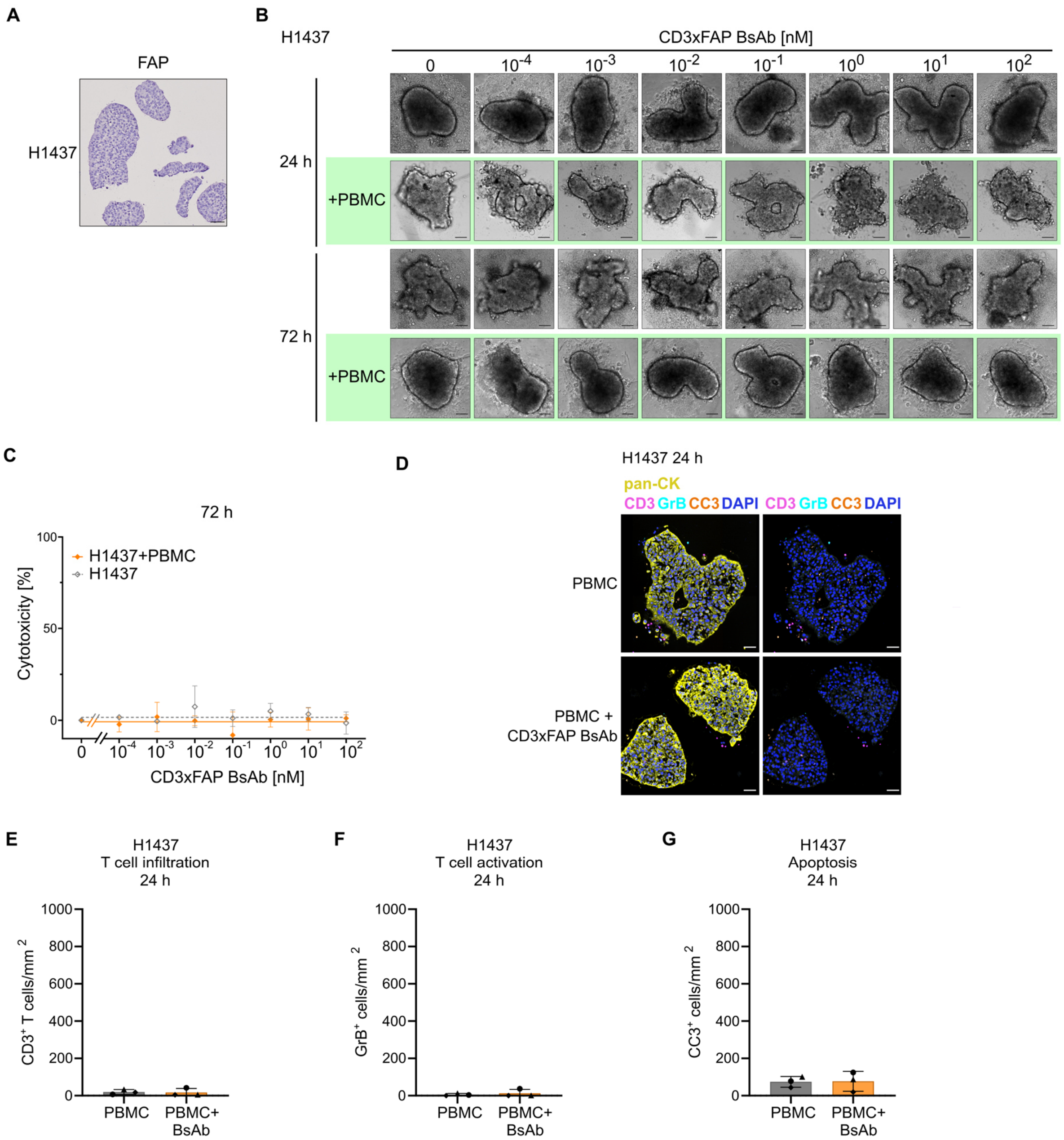

Fig. S4. FAP-negative H1437 spheroids do not respond to CD3xFAP BsAb (OMTX305) treatment. (**A**) FAP expression in H1437 spheroids shown by IHC stainings. Scale bar represents 50 µm. (**B-G**) H1437 spheroids were co-cultured with allogeneic PBMCs and CD3xFAP BsAb (OMTX305)-treated. (**B**) Brightfield images showing the structure of H1437 spheroids treated with increasing BsAb concentrations. Scale bar, 200 µm. (**C**) BsAb-induced cytotoxicity in H1437 spheroids with or without PBMC co-culture, determined by lactate dehydrogenase quantification in the spheroid medium (n=3, mean ± SD). (**D**) T cell infiltration, T cell activation and apoptosis induction in H1437 spheroids co-cultured with PBMCs and treated with 1 nM CD3xFAP BsAb (OMTX305) for 24 h shown by mIF staining of nuclei (DAPI, blue), tumor cells (pan-CK, yellow), T cells (CD3, pink), T cell activation (GrB, cyan) and apoptosis CC3, orange). Scale bar, 50 µm. (**E-G**) (**E**) T cell density (CD3), (**F**) T cell activation (GrB) and (**G**) apoptosis induction (CC3) in H1437 spheroids quantified from mIF stainings (n=3, mean of 10 spheroids per experiment ± SD).

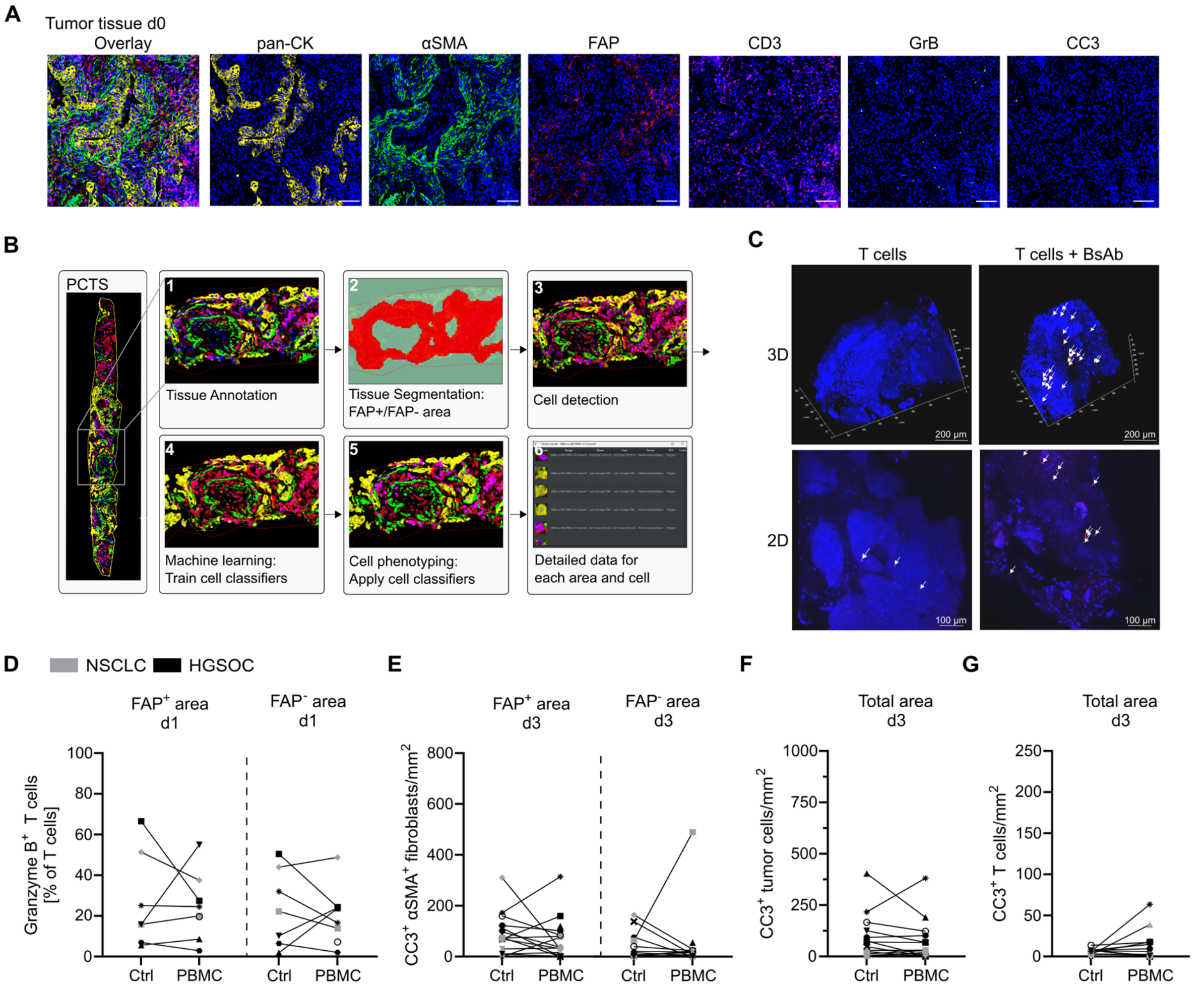

Fig. S5: Image-based assessment of individual drug responses in PCTS. (**A**) Panel used for mIF analysis of CD3xFAP BsAb (OMTX305) treatment response in PCTS staining tumor cells (pan-CK, yellow), fibroblasts (αSMA, green; FAP, red), T cells (CD3, pink), T cell activation (GrB, cyan), apoptosis (CC3) and nuclei (DAPI, blue). Scale bar, 100 µm. (**B**) Workflow of QuPath-based mIF image analysis. (**C**) T cell infiltration in PCTS co-culture with T cells and CD3xFAP BsAb (OMTX305) treatment shown by confocal microscopy. Arrows indicate infiltrated T cells. Scale bar, 200 or 100 µm as indicated. (**D-G**) Quantification of mIF stainings of PCTS (without OMTX305) co-cultured with (PBMC) or without (Ctrl) autologous PBMCs (**D**) T cell activation (%GrB$^+$ cells of CD3$^+$ T cells) in FAP$^+$ or FAP$^-$ areas, (**E**) CC3$^+$ fibroblast density (CC3$^+$ αSMA$^+$) in FAP$^+$ and FAP$^-$ areas and (**F**) CC3$^+$ tumor cell density (CC3$^+$ pan-CK$^+$) or (**G**) CC3$^+$ T cell density (CC3$^+$ CD3$^+$) in the total area. Different patients are indicated by different symbols.

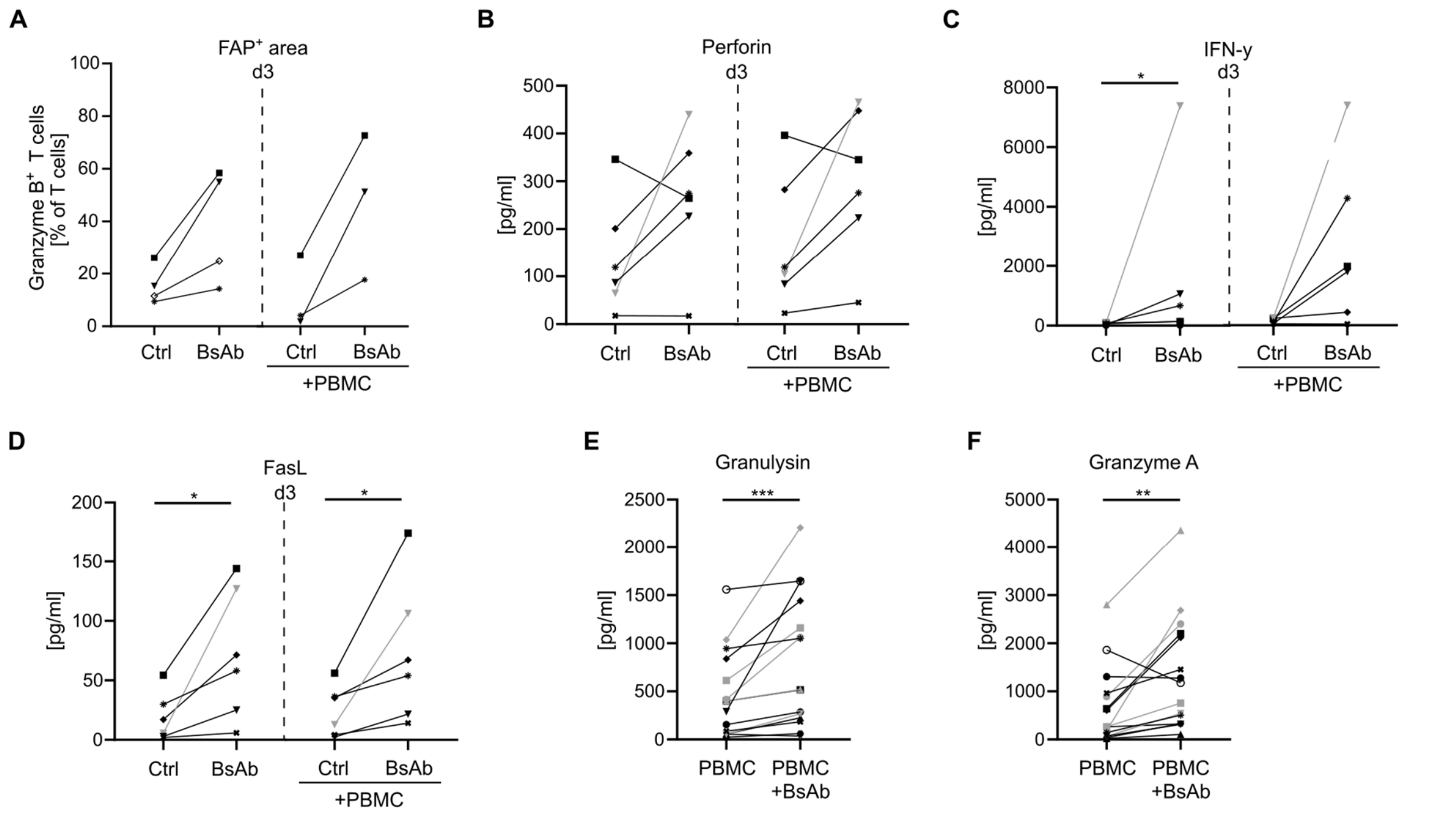

Fig. S6: Cytolytic cytokines are produced in PCTS treated with CD3xFAP BsAb (OMTX305). (**A-F**) PCTS (total n=15) of NSCLC (n=5) or HGSOC (n=10) tumors with or without co-culture with autologous PBMCs and treatment with 100 nM CD3xFAP BsAb (OMTX305) for up to three days. Different patients are indicated by different symbols. (**A**) T cell activation (%GrB$^+$ cells of CD3$^+$ T cells) in the FAP$^+$ area after one day of treatment with or without PBMC co-culture quantified from mIF stainings (n=4). (**B-D**) Levels of (**B**) Perforin, (**C**) Interferon-γ (IFN-γ) and (**D**) Fas ligand (FasL) in PCTS culture supernatants after three days of OMTX305 treatment with or without PBMC co-culture (n=6). (**E**) Granulysin and (**F**) Granzyme A levels in PCTS culture supernatants after three days of PBMC co-culture and OMTX305 treatment (n=15) (Wilcoxon signed-rank test; * p≤ 0.05, ** p≤ 0.01, *** p ≤ 0.001).

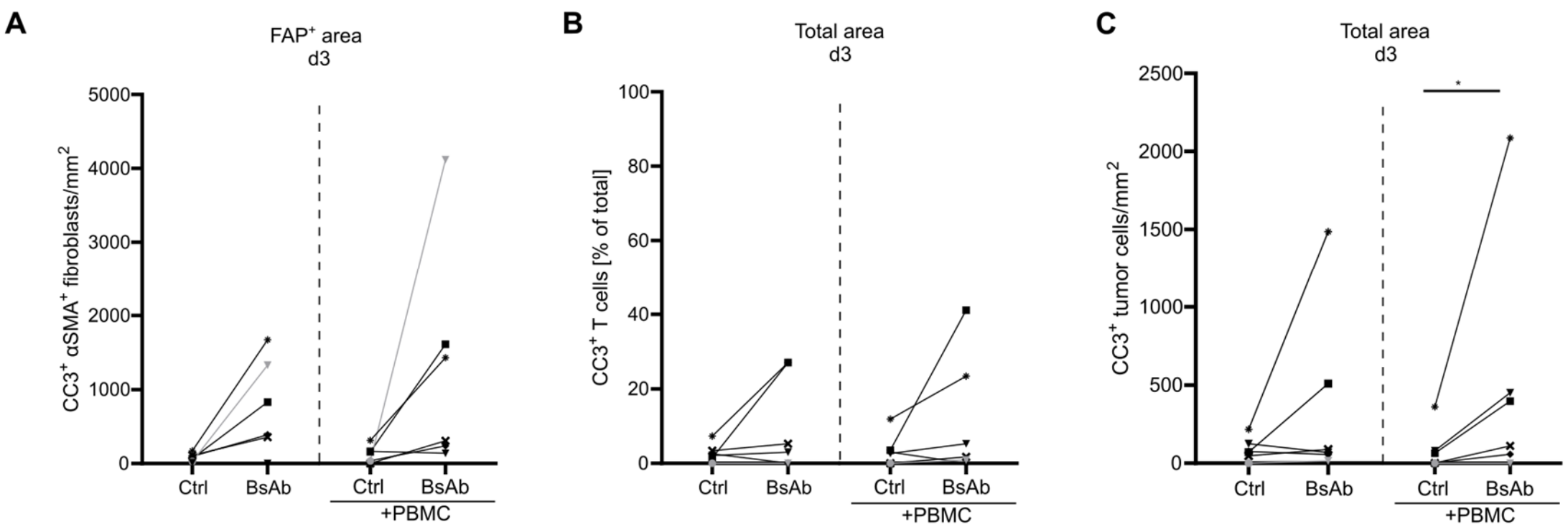

Fig. S7: Cell death induction in fibroblasts, T cells and tumor cells in response to CD3xFAP BsAb with or without PBMC co-culture. (**A-C**) PCTS (total n=6) of NSCLC (n=1) or HGSOC (n=5) tumors with or without co-culture with autologous PBMCs and treatment with 100 nM CD3xFAP BsAb (OMTX305) for up to three days. Different patients are indicated by different symbols. Quantifications of mIF stainings indicating (**A**) $CC3^+$ fibroblast density ($CC3^+$ αSMA^+) in the FAP^+ area, (**B**) percentage of $CC3^+$ T cells (%$CC3^+$ of $CD3^+$ T cells) in the total area and (**B**) $CC3^+$ tumor cell density ($CC3^+$ pan-CK^+) in the total area after three days of treatment (Wilcoxon signed-rank test * $p \leq 0.05$).

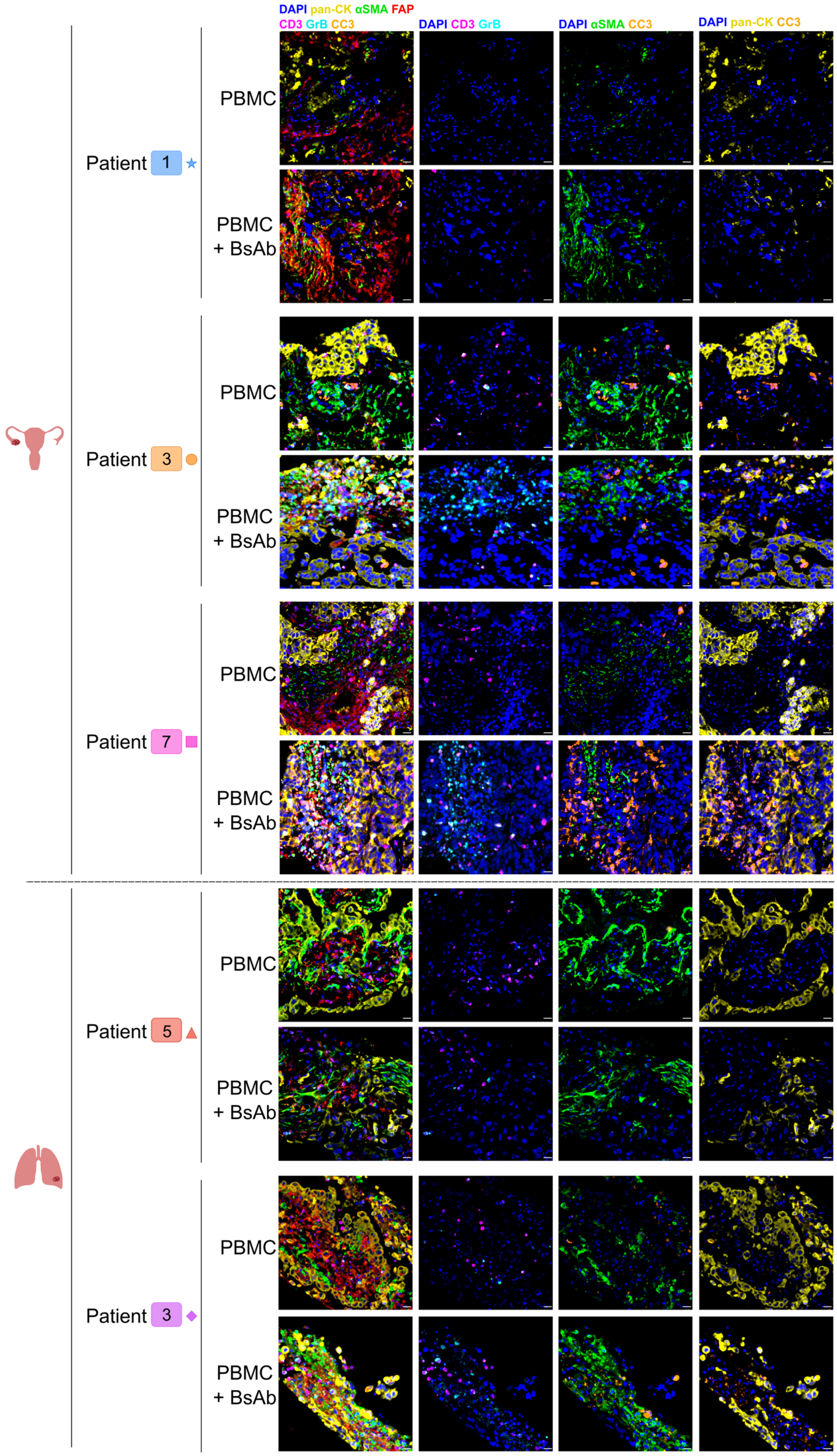
DAPI pan-CK αSMA FAP
CD3 GrB CC3
DAPI CD3 GrB
DAPI αSMA CC3
DAPI pan-CK CC3
Patient 1
PBMC
PBMC + BsAb
Patient 3
PBMC
PBMC + BsAb
Patient 7
PBMC
PBMC + BsAb
Patient 5
PBMC
PBMC + BsAb
Patient 3
PBMC
PBMC + BsAb

Fig. S8: Representative CD3xFAP BsAb (OMTX305) treatment responses in PCTS of individual patients. Granzyme B (GrB) expression and cell death induction in PCTS-PBMC co-cultures treated with CD3xFAP BsAb (OMTX305). PCTS of HGSOC or NSCLC tumors representative for different responses to CD3xFAP BsAb detected by mIF staining of tumor cells (pan-CK, yellow), fibroblasts (αSMA, green; FAP, red), T cells (CD3, pink), T cell activation (GrB, cyan), apoptosis (CC3, orange) and nuclei (DAPI, blue). While HGSOC-patient 1 showed no T cell infiltration, HGSOC-patient 3 displayed elevated GrB levels without a corresponding increase in CC3. HGSOC-patient 7 showed an apoptotic response, with an increase in both $CC3^{+}$ fibroblasts and tumor cells. A non-responsive phenotype was observed in NSCLC-patient 5, which exhibited neither GrB nor CC3 induction. NSCLC-patient 3 on the other hand displayed strong GrB induction alongside apoptosis induction in both stromal and tumor areas. Scale bar, 20 μm.

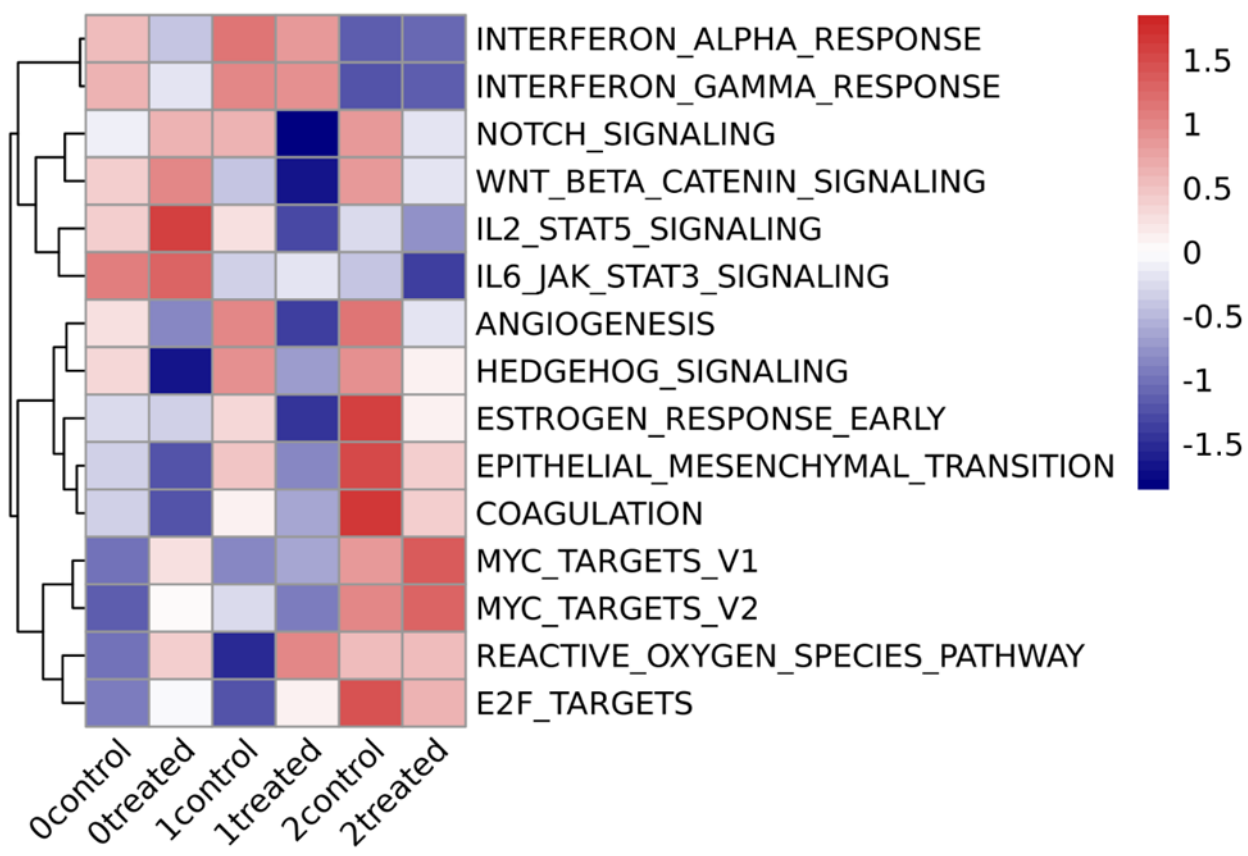

Fig. S9: Gene set variation analysis of BsAb-treated vs. control PCTS in T cell subclusters. T cells from control and CD3xFAP BsAb (OMTX305)-treated PCTS of four HGSOC patients (Patients 1, 2, 3 and 9) were analyzed by single-cell RNA-seq and gene set variation analysis (GSVA) using hallmark signatures from MSigDB.

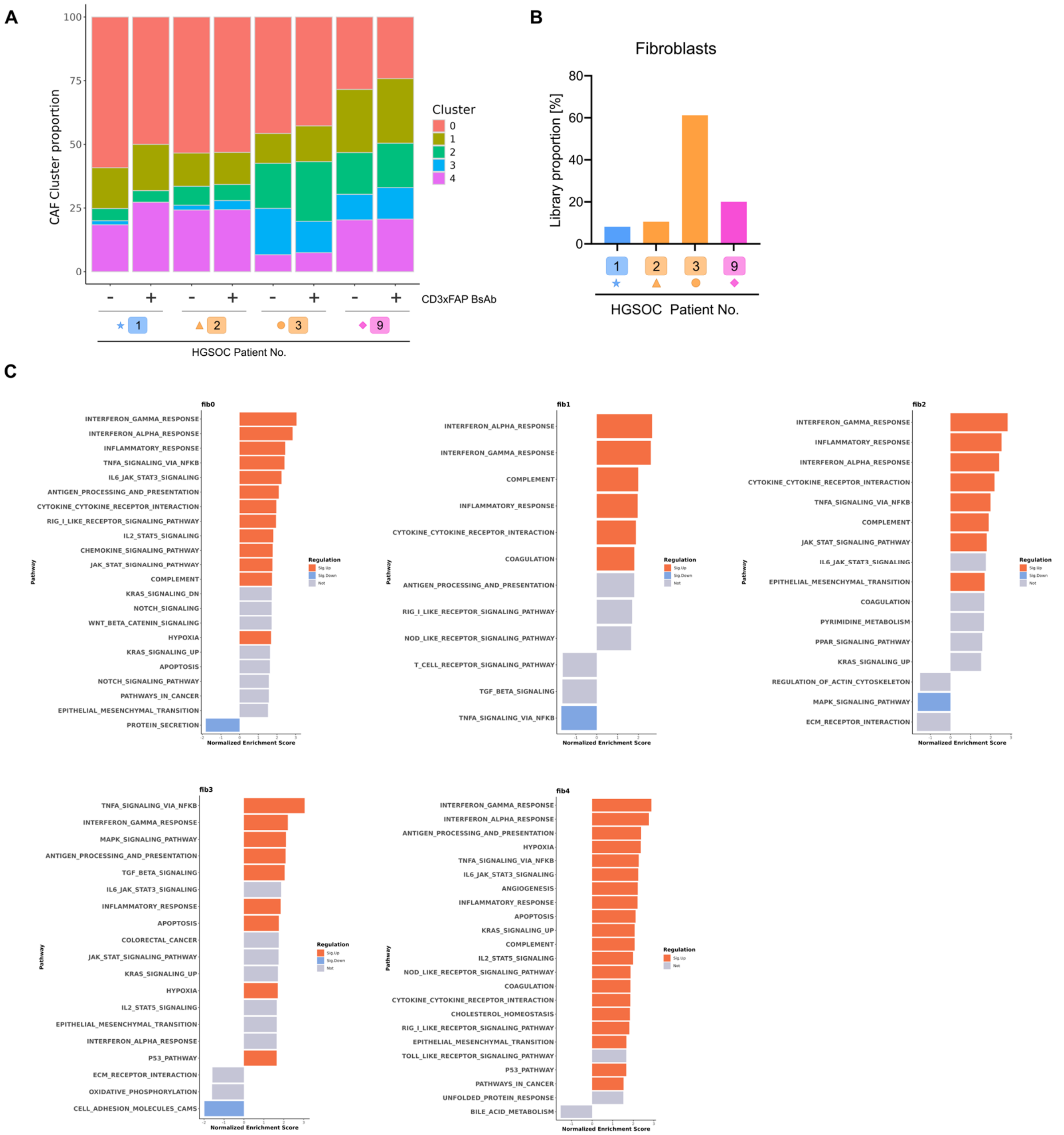

Fig. S10: Single-cell RNA-seq data of CAF subpopulations in PCTS from four HGSOC patients. (**A-C**) Control and CD3xFAP BsAb (OMTX305)-treated PCTS of four HGSOC patient (Patients 1, 2, 3 and 9) were analyzed by single-cell RNA-seq. (**A**) Proportion of CAF subclusters in untreated and BsAb-treated PCTS of different patients. (**B**) Contribution of different patients to the fibroblast library. (**C**) Gene set enrichment analysis of BsAb-treated vs. control PCTS in CAF subclusters.

Patient	Age	TNM-Stage	FIGO	Pre-treatment	CA 125 [U/ml]	BRCA	HRD
1	69	pT3c, L0, V0, Pn0, pM1a, R0	IV A	-	136	n.A.	n.A.
2	81	pT3c, pN0, L1, V1, Pn0	III C	-	190	wt	-
3	72	cT3c, G3, cN1, M1 (HEP) ypT2a, L0, V0, Pn0, R0	IV B	-	n.A.	wt	-
4	64	G3 R2	IV B	-	1782	wt	-
5	87	ypT1c3, L1, V1, Pn0	III C	Relapse after 6xCb/Pac + Beva + Olaparib maintenance	54	BRCA1mut, BRCA2 wt	n.A.
6	56	pT3c, pN1b (1/1), pM1b, L1, V0, Pn0, R1	IV B	-	768	BRCA1 wt, BRCA2mut	n.A.
7	81	T3c, ER 8/12, PR 1/12	III C	-	2251	wt	-
8	74	pT3b pN1b (2/3) L1 V1 Pn0 cM1 (LK) R2 (Jejenum)	IV B	-	778	wt	-
9	65	ypT3b, L0, V0, Pn0, R0	III C	-	1001	BRCA1 wt, gBRCA2mut	+
10	62	pT3c, pN1b (2/6), L1, V1, Pn0	III C	-	360	wt	+

Table S1: HGSOC Patient information. All samples were derived from female HGSOC patients. CA125 values were measured before surgical resection. Beva, Bevacizumab; BRCA, breast cancer gene; CA, cancer antigen; Cb, carboplatin; HRD, homologous recombination deficiency; n.A., not available; Pac, paclitaxel; wt, wildtype.

Patient	Age	Gender	TNM-Stage	Pre-treatment	% PD-L1$^+$ in tumor cells	TTF	Mutation
1	77	female	pT2b, pN1 (1/31), L1, V0, Pn0, R0, G2	-	0	+	EGFR E709K+G719S; activating
2	68	male	pT2b, pN0, L0, V0, Pn0, R0, G3	-	1-2	+	KRAS p.G12V activating
3	81	female	pT4, pN0, L0, V0, Pn0, R0, G3	-	15	-	KRASp.G12C; TP53 p.P278R
4	53	female	pT2a, pN0 (0/17), L0, V0, Pn0, R0, G3	-	30	+	-
5	61	male	pT2b, pN1 (7/28), L1, V0, Pn0, R0, G2	-	100	n.A.	-

Table S2: NSCLC Patient information. All samples were derived from treatment-naïve NSCLC adenocarcinomas. EGFR, epidermal growth factor receptor; KRAS, Kirsten rat sarcoma virus; PD-L1, programmed death-ligand 1; TP53, tumor protein p53; TTF, thyroid transcription factor.

Molecule	Composition	Function
OMTX005	Anti-FAP IgG1	FAP-binding antibody
OMTX305	2+1 (Fab-eIg) FAPxCD3 BsAb	FAPxCD3 BsAb
OMTX705	Anti-FAP IgG1 OMTX005 + cytolysin TAM558	FAP-ADC, recognizes the same FAP epitope as OMTX305
OMTX703-mu	Anti-murine ADC	Negative control, does not cross-react with human tissue
Secondary antibody	Anti-TAM558 polyclonal antibody	Secondary antibody to detect binding of anti-FAP IgG1 of OMTX705 to tissue sections

Table S3: Molecules used for cross-reactivity studies of OMTX305.

	Patient	Distance to FAP$^+$ area (mean±SD) [µm]			
		PBMC d3		PBMC+BsAb d3	
		CC3$^-$ tumor cells to FAP$^+$ area	CC3$^+$ tumor cells to FAP$^+$ area	CC3$^-$ tumor cells to FAP$^+$ area	CC3$^+$ tumor cells to FAP$^+$ area
HGSOC	1	8.15 (±15.38)	9.73 (±12.06)	48.60 (±57.17)	no CC3$^+$ tumor cells detected
	2	73.27(±55.54)	88.28 (±63.02)	131.74 (±138.3)	96.62 (±106.46)
	3	21.09 (±25.81)	18.58 (±23.31)	15.08 (±18.03)	10.77 (±18.02)
	4	16.11 (±19.38)	no CC3$^+$ tumor cells detected	18.87 (±22.83)	15.25 (±19.33)
	5	0 (±0)	20.28 (±2.04)	12.29 (±11.42)	23.16 (±21.03)
	6	28.02 (±25.58)	31.43 (±30.04)	61.20 (±43.01)	45.77 (±39.82)
	7	6.46 (±12.88)	5.14 (±13.46)	25.03 (±20.04)	13.14 (±15.46)
	8	33.38 (±34.11)	30.97 (±29.37)	no tumor cells detected	
	9	103.42 (±56.09)	98.20 (±63.44)	44.09 (±16.67)	24.64 (±30.66)
	10	15.10 (±18.63)	6.16 (±12.23)	41.06 (±35.13)	25.16 (±23.42)
NSCLC	1	61.41 (±66.28)	no CC3$^+$ tumor cells detected	704.56 (±927.34)	753.75 (±146.03)
	2	60.06 (±44.44)	64.20 (±39.35)	30.70 (±23.84)	10.91 (±15.68)
	3	34.02 (±51.58)	50.66 (±68.08)	25.80 (±34.12)	33.18 (±34.81)
	4	315.05 (±323.39)	462.90 (±404.95)	622.11 (±267.35)	717.83 (±256.38)
	5	9.68 (±12.17)	12.12 (±16.82)	21.64 (±33.22)	15.58 (±14.24)

Table S4: Distances of non-apoptotic and apoptotic tumor cells to the FAP$^+$ area. Tumor slices were co-cultured with PBMCs with or without CD3xFAP BsAb (OMTX305) for three days. Mean distances between CC3$^-$ tumor cells (pan-CK$^+$ cells) or CC3$^+$ tumor cells to the FAP$^+$ area in µm were quantified from multiplex immunofluorescence stainings. Data was generated from analysis of whole tumor slice cross-sections of each patient.
